# PRH1 mediates ARF7-LBD dependent auxin signaling to regulate lateral root development in *Arabidopsis thaliana*

**DOI:** 10.1371/journal.pgen.1008044

**Published:** 2020-02-07

**Authors:** Feng Zhang, Wenqing Tao, Ruiqi Sun, Junxia Wang, Cuiling Li, Xiangpei Kong, Huiyu Tian, Zhaojun Ding

**Affiliations:** The Key Laboratory of the Plant Cell Engineering and Germplasm Innovation, Ministry of Education, School of Life Sciences, Shandong University, Qingdao, Shandong, China; Wake Forest University, UNITED STATES

## Abstract

The development of lateral roots in *Arabidopsis thaliana* is strongly dependent on signaling directed by the AUXIN RESPONSE FACTOR7 (ARF7), which in turn activates LATERAL ORGAN BOUNDARIES DOMAIN (LBD) transcription factors (*LBD16*, *LBD18 and LBD29*). Here, the product of *PRH1*, a *PR-1* homolog annotated previously as encoding a pathogen-responsive protein, was identified as a target of ARF7-mediated auxin signaling and also as participating in the development of lateral roots. *PRH1* was shown to be strongly induced by auxin treatment, and plants lacking a functional copy of *PRH1* formed fewer lateral roots. The transcription of *PRH1* was controlled by the binding of both ARF7 and LBDs to its promoter region.

## Author summary

In *Arabidopsis thaliana* AUXIN RESPONSE FACTOR7 (ARF7)-mediated auxin signaling plays a key role in lateral roots (LRs) development. The *LATERAL ORGAN BOUNDARIES DOMAIN (LBD)* transcription factors (*LBD16*, *LBD18* and *LBD29*) act downstream of ARF7-mediated auxin signaling to control LRs formation. Here, the *PR-1* homolog *PRH1* was identified as a novel target of both ARF7 and LBDs (especially the LBD29) during auxin induced LRs formation, as both ARF7 and LBDs were able to bind to the *PRH1* promoter. This study provides new insights about how auxin regulates lateral root development.

## Introduction

The architecture of the root system depends on the density of lateral roots (LRs) formed along with the extent of root branching. LRs are initiated from mature pericycle cells lying adjacent to the xylem pole, referred to in *Arabidopsis thaliana* as the xylem pole pericycle [1, 2]. A subset of these cells, namely the founder cells, undergo a series of highly organized divisions to form an LR primordium, which eventually develops into an LR. The entire process of LR development, from its initiation to its emergence, is regulated by auxin [1, 3, 4]. The accumulation of auxin in protoxylem cells results in the priming of neighboring pericycle cells to gain founder cell identity, forming an LR pre-branch site [5]. This auxin signaling is mediated by the proteins ARF7 and ARF19, which act in the IAA14/28-dependent pathway [6, 7]. LR initiation depends on the transcriptional activation, through the involvement of the transcription factor *LBD16* and *LBD18*, of either *E2Fa* or *CDKA1;1* and *CYCB1;1* [8, 9]. Other downstream targets such as EXP14 and EXP17 and the products of certain cell wall loosening-related genes are also either directly or indirectly regulated by LBD18 to promote the emergence of an LR [10–12]. LBD18 has been also found to control LR formation through its interaction with GIP1, which results in the transcriptional activation of *EXP14 [13]*.

LBD transcription factors are among the most well studied downstream targets of ARF7 and ARF19 [14]. ARF7 has also been found to control LR emergence through its regulation of *IDA* and genes which encode leucine-rich repeat receptor-like kinases such as *HAE* and *HSL2* [15]. These interactions take place in the overlaying tissues, thereby influencing cell wall remodeling and cell wall degradation during LR emergence [15].

Here, the product of *PRH1* (*At2g19990*), a homolog of *PR-1* which has been annotated as a pathogen responsive protein [16], was identified as a novel target of ARF7-LBDs mediated auxin signaling. We provided evidence that PRH1 acted downstream of ARF7 and LBDs to control auxin-regulated LR formation.

## Results

### PRH1 is a target of ARF7-mediated auxin signaling

**Fig 1:**
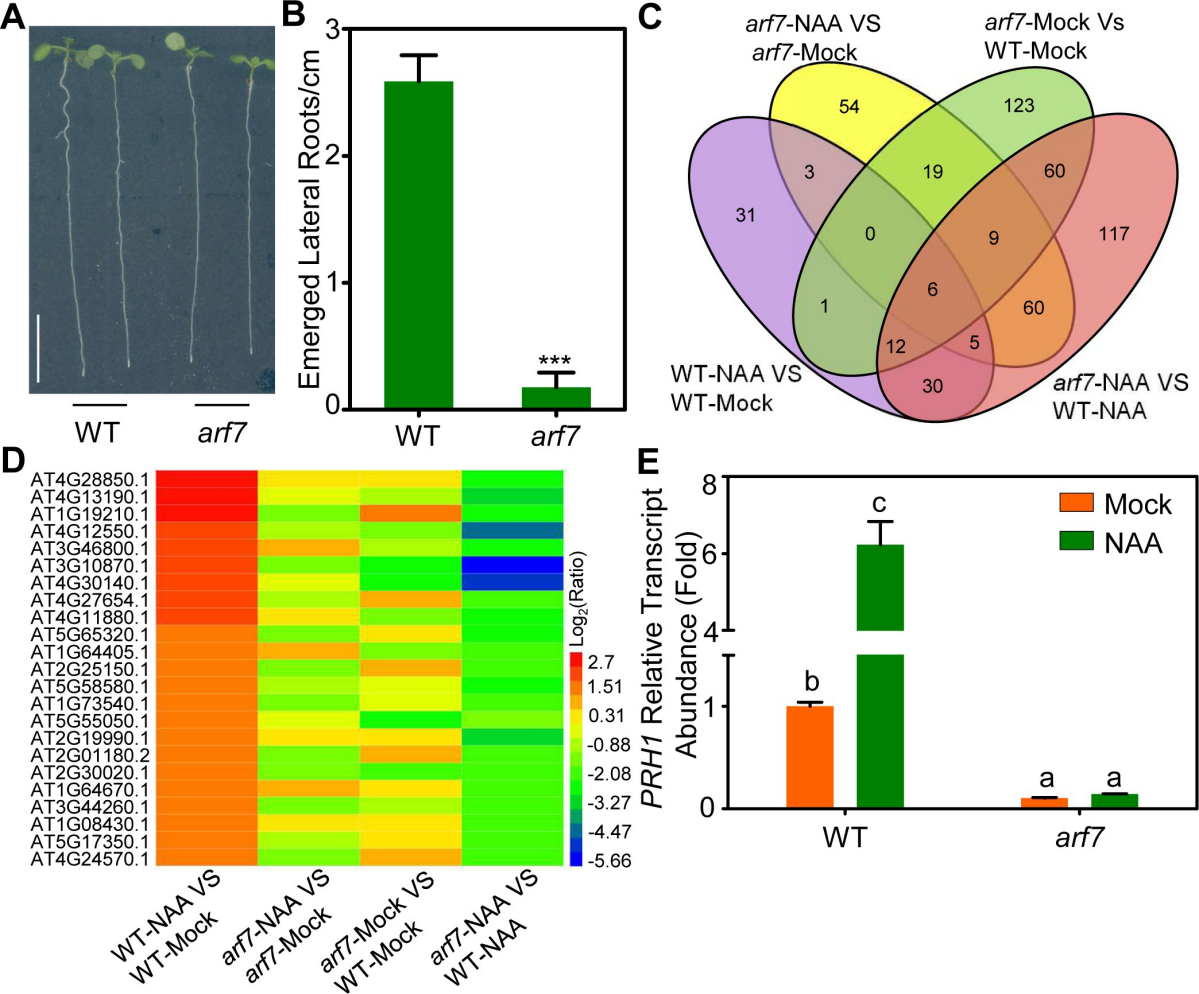
*PRH1* is a downstream target of ARF7-mediated auxin signaling. (A) Eight-day-old seedlings of WT and *arf7*. Scale bar: 1 cm. (B) The ratio between the number of the emerged lateral roots and the length of the primary root of WT and *arf7* seedlings. Data shown as mean±SE, three biological replicates in the experiment, 20 plant seedlings for each repeat. ***: the mutant’s performance differs significantly (*P*<0.001) from that of WT. (C) Venn diagram indicating the overlap of differentially expressed genens between the four datasets (Col-NAA VS Col-Mock, *arf7*-NAA VS *arf7*-Mock, *arf7*-Mock VS Col-Mock and *arf7*-NAA VS Col-NAA) based on RNA-seq. RNA-seq analysis: RNA was extracted from the primary roots of eight-day-old seedlings exposed or not exposed to 10 μM NAA for 4 hours. NAA: naphthalene acetic acid. (D) Heat map of the differentially expressed genes induced by auxin and regulated by ARF7 according to RNA-seq data. (E) The relative transcript abundance of *PRH1* in the roots of eight-day-old WT and *arf7* seedlings exposed (green) or not exposed (orange) to 10 μM NAA for 4 hours. The relative transcript abundance is relative to the untreated WT. Values represent averages of three biological replicates in the experiment, and the total mRNA was extracted from about 100 seedlings for each repeat. Error bars represent SE. Different letters atop the columns indicate significant (*P*<0.001) differences in abundance.

To identify additional targets involved in ARF7-mediated auxin signaling and LR development (Fig 1A and 1B), a comparison was made between the 8-day-old wild type (WT) or *arf7* mutant seedlings exposed to 10 μM naphthalene acetic acid (NAA) for 4 hours respectively (Figs 1C and S1A). Based on differential comparisons (WT and *arf7* with or without NAA treatment) all of the detected genes were presented in S1 Table as the raw data and the genes changed at least 2-fold (*P* < 0.0001, log_2_fold change >1) were selected as differentially expressed genes (DEGs) shown in S2 Table. The DEGs were selected based on the comparisons (WT-NAA VS WT-Mock, *arf7*-NAA VS *arf7*-Mock, *arf7*-Mock VS WT-Mock, *arf7*-NAA VS WT-NAA), which were induced by auxin and regulated by ARF7. To narrow down the selection of candidate genes, we further adjusted the screening criteria (*P* < 0.0001, log_2_fold change >1.5 in WT-NAA VS WT-Mock), which reduces the DEGs to 23 (Fig 1D and S1B Fig). To further explore whether these genes are involved in LR development, we then examined the expression patterns of these 23 candidate genes in LR initiation using the *Arabidopsis* eFP Browser website (http://bar.utoronto.ca/efp/cgi-bin/efpWeb.cgi). After this analysis, eight of the candidate DEGs were expressed in the process of LR initiation induction, and then we focused on the *PRH1* (At2g19990), which displayed the highest induction by auxin while the induction was absent in *arf7*, for further investigations (Fig 1E, S1B and S1C Fig).

**Fig 2:**
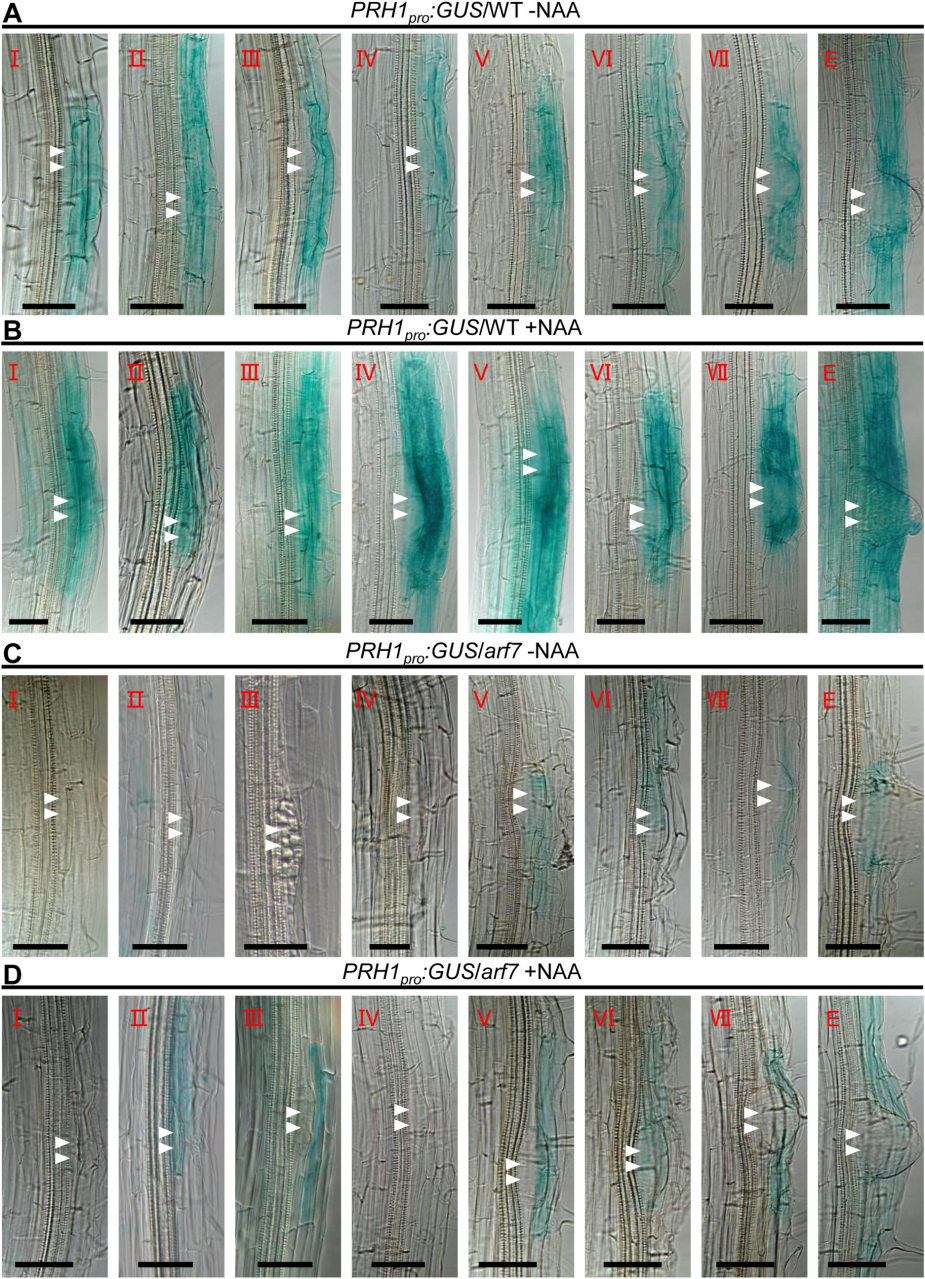
Histological localization and expression of *PRH1pro*:*GUS* transgene during LR formation. (A) and (B) *PRH1* is expressed in the cells overlying/surrounding the lateral root primordium(LRP) in WT seedlings not exposed to auxin(A), and the intensity of its expression is strongly enhanced when the plants were treated with 10 μM naphthalene acetic acid (NAA) for 4 hours (B). (C) and (D) GUS activity is suppressed in non-treated *arf7* seedlings (C), and even more so in treated ones (D). NAA: naphthalene acetic acid. Before GUS staining the seedlings were soaked for 4 hours in liquid half strength Murashige and Skoog (MS) medium, which containing 10 μM NAA or not. The developmental stages (I through VII and E) of the LRs are indicated in the top left corner. GUS (β-glucuronidase) signals are shown in blue. Stages I to VII of primordia were based on the classification by Malamy and Benfey [1], E: Emerged lateral roots, Scale bars: 50 μm.

*PRH1*, a homolog of *PR-1* has been annotated as encoding a pathogen-responsive protein and was also named as PR-1-LIKE [16]. There are 11 members in the family in *Arabidopsis*, and most of them are defense-related genes (S2A Fig) [17–19]. Expression of *PRH1* is not salicylic acid responsive and the biochemical function is unknown [16]. Considering that *PRH1* was induced by auxin (Fig 1E), the auxin induced *GH3*.*5* was used as a positive control and the *PRH1* expression was examined through time-coursed treatment with auxin (treated with 10 μM naphthalene acetic acid, NAA). The result showed that *PRH1* is transcriptionally regulated by auxin and there was a significant induction after 2 hours of auxin treatment (S2B Fig). The *GUS* expression analysis in 8-day-old seedlings carrying a *PRH1pro*:*GUS* transgene implied that *PRH1pro*:*GUS* is expressed in the cells overlying/surrounding the lateral root primordium (LRP), especially at endodermis or cortex cells overlying the primordium in WT background (Fig 2A; S2C Fig), and the level of GUS activity was greatly enhanced following exposure of the plants to auxin (Fig 2B). When the *PRH1pro*:*GUS* transgene was expressed in the *arf7* mutant background, the *PRH1pro*:*GUS* expression was strongly suppressed, and auxin treatment only slightly induced the expression of *PRH1pro*:*GUS* (Fig 2C and 2D). Consistently, *PRH1* expression was completely non-responsive to auxin treatment in *arf7* according to our qPCR assays (Fig 1E). The conclusion was that the auxin-responsive *PRH1* is mainly expressed in the cells overlying/surrounding the LRP and its expression is dependent on ARF7-mediated auxin signaling.

### PRH1 acts downstream of ARF7 to regulate LR development

**Fig 3:**
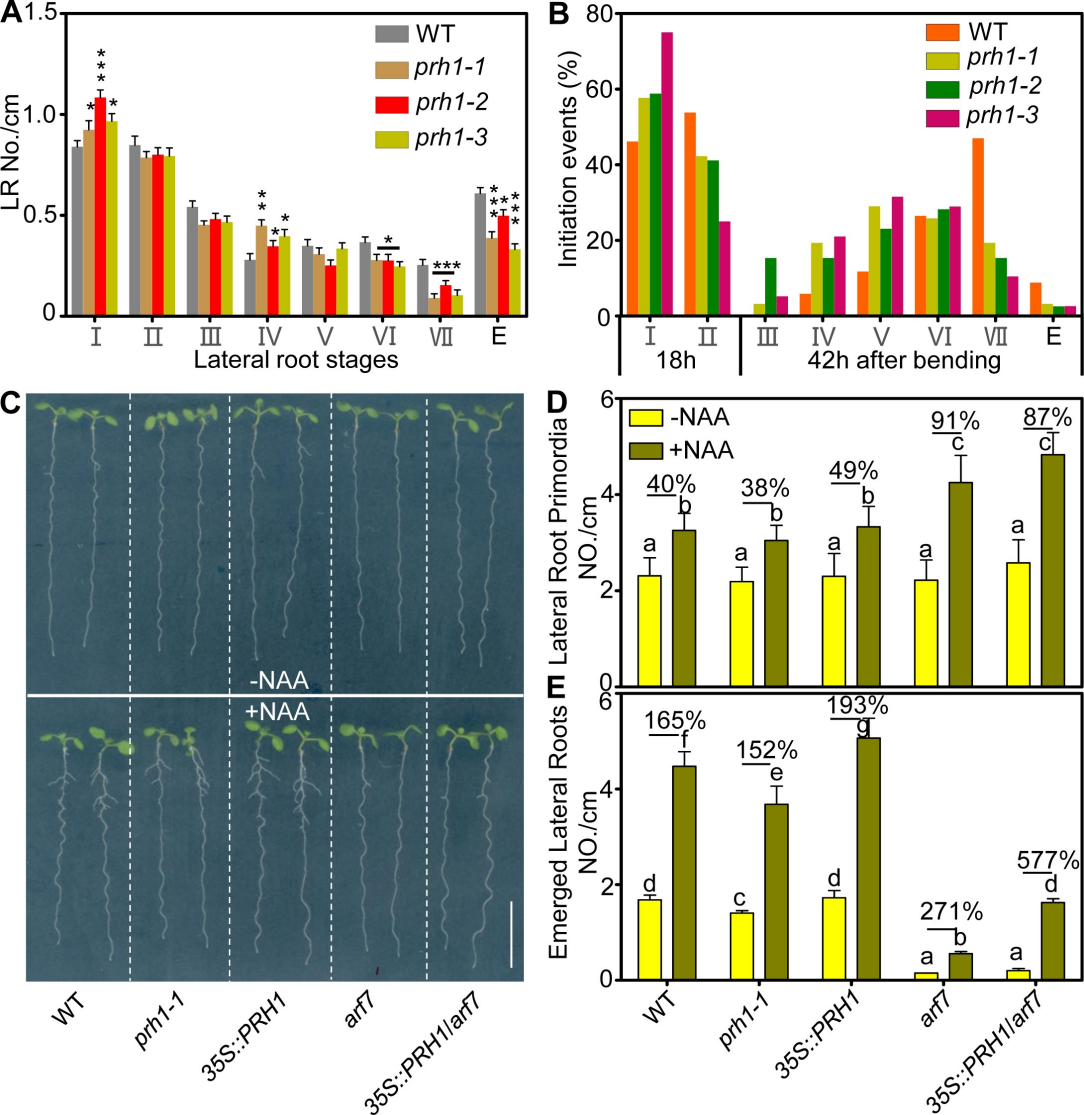
*PRH1* acts downstream of ARF7-mediated lateral root (LR) development. (A) Density of primordia at given stages. Stages I to VII of primordia were based on the classification by Malamy and Benfey [1], E: emerged lateral roots, *,**, ***: means differ significantly (P<0.05, P<0.01, P<0.001) from the WT control. (B) The synchronized initiation of lateral root primordia were induced using gravitropic stimulation at the site of root bending in the 3-day-old seedlings of WT, *prh1-1*, *prh1-2* and *prh1-3*. Phenotypic analysis of lateral root (LR) emergence was achieved after 18 h and 42 h gravistimulus compared with WT (Col-0) and the LR emergence is delayed in *prh1* mutants. Lateral root primordium stages (from I to VII according to previous descriptions from 1997 Benfey [1]) were analyzed from 30 seedlings. (C) Eight-day-old seedlings of WT, *arf7*, *prh1-1* mutants and transgenic lines harboring either *35S*::*PRH1* or *35S*::*PRH1/arf7*. The upper panel shows the seedlings grown on 1/2 MS medium without NAA addition and the lower panel shows the seedlings grown on 1/2 MS medium containing 30 nM NAA. Bar: 1 cm. (D and E) Density of lateral root primordia (D) and emerged lateral roots (E) of the 8-d-old seedlings grown on the half strength Murashige and Skoog (MS) medium containing 30 nM NAA or not. The percentage values represent the increase with NAA treatment. The density was the ratio between lateral roots number and the length of primary root. NAA: naphthalene acetic acid. Different letters atop the columns indicate significant (*P*<0.05) differences in abundance. Data shown as means±SE, three biological replicates in the experiment, 20 plant seedlings for each repeat.

To address if auxin-induced *PRH1* expression is involved into LR development, we examined the LR phenotypes in both *prh1* mutants and the *PRH1* over-expression lines (Fig 3A and 3B; S3B Fig). The third allele of *prh1* (*phr1-3*) generated via Crispr-cas9 system [20] was also used for the LR phenotype analysis (S3A Fig). The primordia numbers of 8-day-old seedlings at various stages were counted of all these three *prh1* mutant alleles. The results showed that primordium development was affected. The numbers at stage I and Ⅳ were increased and at the later periods (stage Ⅵ, Ⅶ and emerged) were reduced (Fig 3A). Examination of LR development in *prh1* mutants showed that the absence of a functional copy of *PRH1* inhibited the formation of emerged LRs (Fig 3E). The expression level of *PRH1* in its T-DNA insertion mutants and the over-expression lines were all verified (S3C Fig). The weak phenotype of *prh1-2* in the emerged stage might be result from the down-regulation of *PRH1* in this allele (Fig 3A; S3C Fig). Moreover the *PRH1pro*:*PRH1* construct was transformed in the *prh1-1* mutant, the expression of *PRH1* could complement the LR defects in *prh1-1* (S4 Fig). Though LR development was not greatly affected by the over-expression of *PRH1* in a WT background (S3B Fig), over-expression of *PRH1* could significantly increase the LR numbers of *arf7* mutant plants in the presence of exogenous NAA treatment (Fig 3C–3E).

To further confirm the role of PRH1 in LR formation, we did LR analysis of *prh1* mutants through a gravistimulation-based bioassay [21–23]. After synchronizing LR formation with a gravistimulus for 18 h, we observed a delayed transition from stage I to stage II of LR development which was shown by the increased LR I (lateral root stage I) ratio and decreased LR II (lateral root stage II) ratio in *prh1* mutants (Fig 3B). Then we examined the phenotype of mutant seedlings after a gravistimulus for 42 h, and the lateral root initiation rates reduced both in stage VII and emerged ones, but increased in the earlier stages (stage IV or V) (Fig 3B). In summary, PRH1 acts downstream of ARF7 to regulate LR development.

### ARF7 directly regulates *PRH1* transcription

**Fig 4:**
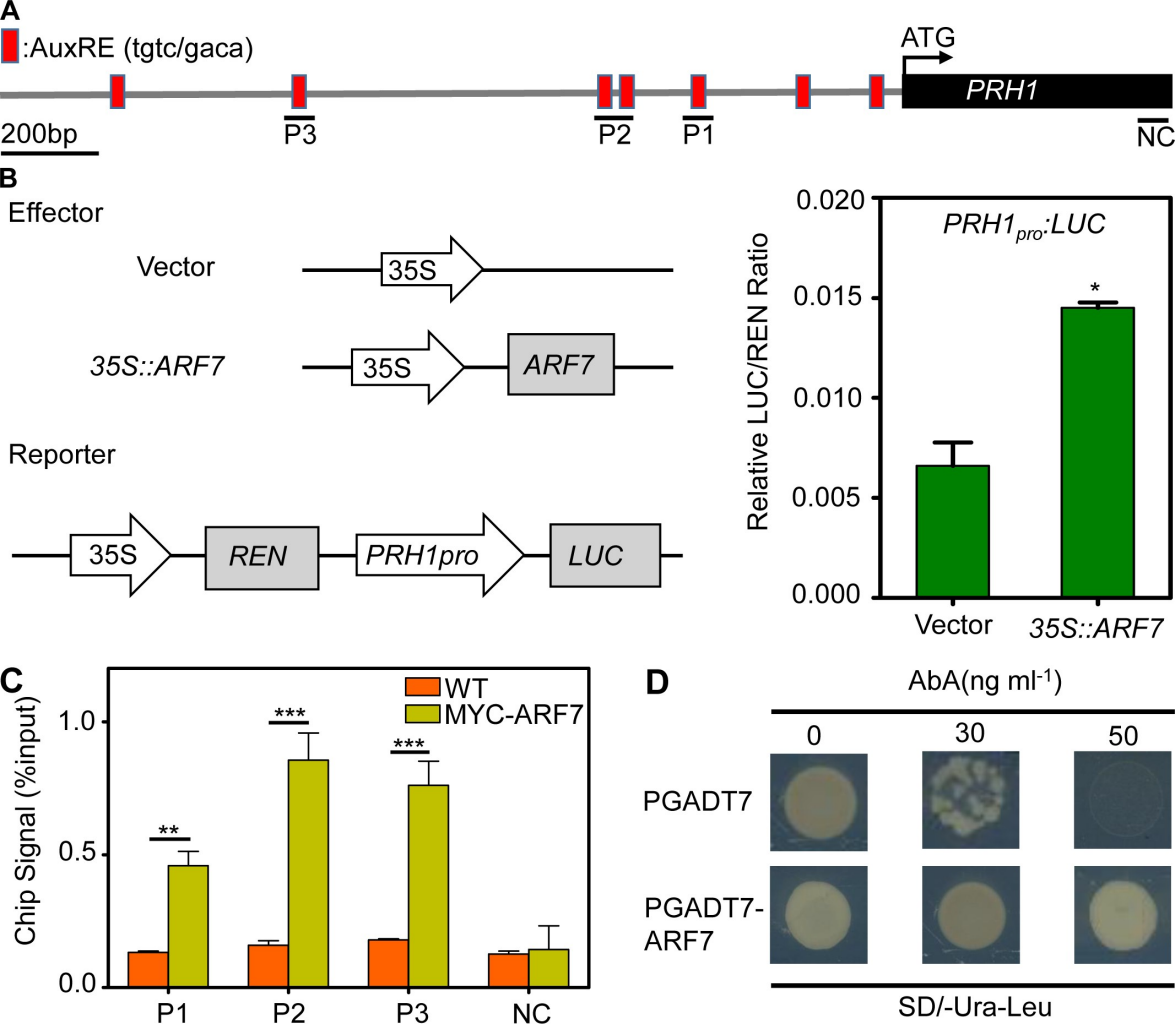
*PRH1* is regulated by *ARF7* at the transcriptional level. (A) Structure of *PRH1* promoter and the fragments used in the CHIP-qPCR assay. AuxREs are indicated by red squares, and black lines show the promoter regions containing the AuxREs used in this assay. NC: negative control. AuxREs: auxin response elements. (B) ARF7 transactivates the *PRH1* promoter in *A*. *thaliana* leaf protoplasts. The left hand panel is a schematic of the effector (*35S*::*ARF7*) and reporter (*PRH1pro*:*LUC*) constructs. The empty vector pBI221 was used as a negative control; the right hand panel shows the ratio of ARF7 drived *LUC* and the empty vector (negative control) to 35S promoter drived *REN* respectively. LUC: firefly luciferase activity, REN: renilla luciferase activity. Values shown as means±SE, three biological replicates in the experiment. *: means differ significantly (*P*<0.05) from the negative control. (C) ARF7 is associated with the *PRH1* promoter according to a CHIP-qPCR assay. Chromatin isolated from a plant harboring *35S*::*MYC-ARF7* and a WT mock control was immunoprecipitated with anti-MYC antibody following the amplification of regions P1, P2 and P3. The coding region segment NC was used as the negative control. The ChIP signal represents the ratio of bound promoter fragments (P1-P3) after immunoprecipitation to total input without immunoprecipitation. Values shown as means±SE, three biological replicates in the experiment. **, ***: means differ significantly (*P*<0.01, *P<*0.001) from the WT control. (D) Physical interaction of ARF7 with the *PRH1* promoter according to a Y1H assay. The plasmid pGADT7-ARF7 was introduced into Y1H Gold cells harboring the reporter gene *PRH1pro*:*AbAr* and the cells were grown on SD/-Ura-Leu medium in the presence of 30 or 50 ng/mL aureobasidin A (AbA). The empty vector pGADT7 was used as a negative control.

The observation that the abundance of *PRH1* transcript was reduced in the *arf7* mutant (Fig 1D) suggested that the gene was transcriptionally regulated by the auxin response factor ARF7. The gene’s promoter sequence harbors the known auxin response elements (AuxREs) TGTC (Fig 4A). A transient expression assay carried out in *A*. *thaliana* leaf protoplasts showed that ARF7 was able to activate a construct comprising the *PRH1* promoter fused with *LUC*. The co-expression of *PRH1pro*:*LUC* and *35S*::*ARF7* had a large positive effect on the intensity of the luminescence signal (Fig 4B), consistent with the idea that ARF7 is able to activate the *PRH1pro*:*LUC* construct. A ChIP-qPCR assay confirmed the association of ARF7 with the AuxRE elements present on the *PRH1* promoter (Fig 4A and 4C), and a yeast one hybrid assay showed that ARF7 was able to bind to the *PRH1* promoter (Fig 4D). The conclusion was that the effect of ARF7 binding to the *PRH1* promoter was regulating the gene’s transcription, thereby influencing LR development.

### Auxin-induced *PRH1* expression is regulated by LBD29

Members of the LBD family are known to function downstream of ARF7 to control LR initiation and emergence [14, 24–27]. When the transcription level of *PRH1* was investigated in the three single *lbd* mutants *lbd16* (Salk_040739), *lbd18* (Salk_038125) and *lbd29* (Salk_071133) combined with or without exogenous auxin treatment, the observation was that the gene was strongly repressed in the single mutants. Though the auxin induced *PRH1* expression was normal in *lbd16* or *lbd18*, the induction was strongly reduced in *lbd29*, indicating LBD29 plays an important role in auxin induced *PRH1* expression (S5A Fig). Consistently, the over-expression of *LBD29* strongly up-regulates *PRH1* expression (S5B Fig). In addition, we also observed the up-regulation of *PRH1* expression in LBD16, LBD18 or LBD29 over-expression lines (S5B Fig). These results suggest that LBDs regulate the transcription of *PRH1*, auxin induced expression of *PRH1* is dependent on LBD29.

Since the auxin influx carrier LIKE-AUXIN3 (LAX3) has been reported to act downstream of ARF7-LBD29 auxin signaling module to facilitate LR emergence [27, 28], we also investigated the relationship between LAX3 and PRH1 through qPCR analysis. The results showed that the absence of PRH1 did not affect the expression of *LAX3* with or without auxin treatment (S5C Fig). However, in *lax3* which has defects in auxin influx, the auxin-induced *PRH1* expression was reduced (S5D Fig).

### LBDs regulate *PRH1* by binding to its promoter

**Fig 5:**
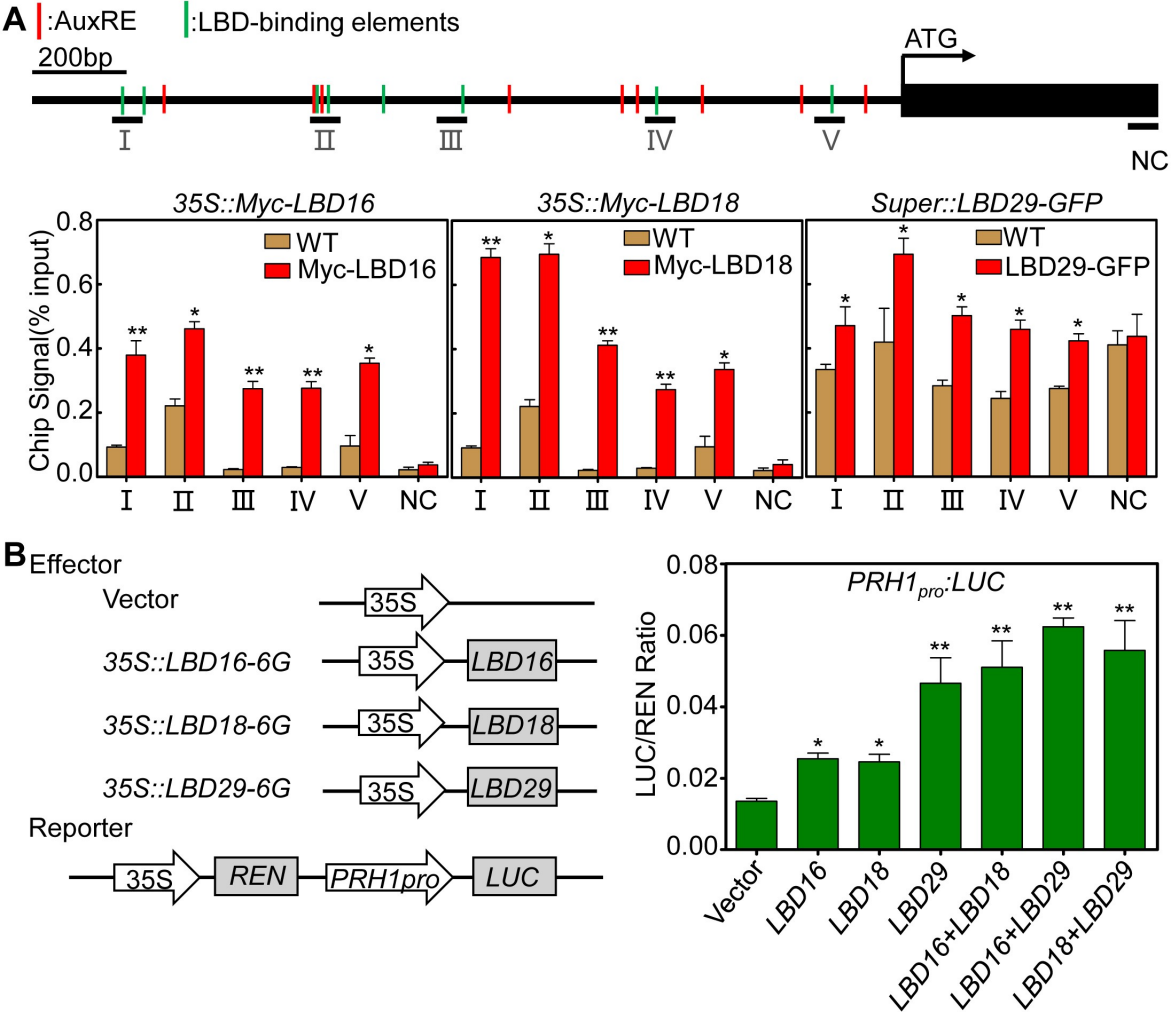
The transcription of *PRH1* is activated by LBD. (A) LBDs associated with the promoter of *PRH1* according to a CHIP-qPCR assay. The upper panel shows the structure of the *PRH1* promoter, and five uniformly distributed sites (black lines) were selected for the CHIP-qPCR assay. Sites II and III include the LBD29 and LBD18 binding motif (See S6 Fig). NC: negative control. The red lines indicate the AuxREs (auxin response elements) and the green lines indicate LBD-binding elements [12, 44]. The lower panel demonstrates the ratio of bound promoter fragments (Ⅰ-Ⅴ) after immunoprecipitation to total input without immunoprecipitation. The samples were derived from WT seedlings (negative control) and those harboring one of the transgenes *35S*::*Myc-LBD16* (Myc-LBD16), *35S*::*Myc-LBD18* (Myc-LBD18), *Super*::*LBD29-GFP* (LBD29-GFP) [44, 49]. Values shown as means±SE, three biological replicates in the experiment. * Represents a comparison of the signal arising from the Myc/GFP-fused LBD with WT in the corresponding genomic region. *, **: means differ significantly (*P*<0.05, *P<*0.01) from the WT control. (B) LBDs transactivate the *PRH1* promoter in *A*. *thaliana* leaf protoplasts as shown by a transient dual-luciferase assay. The reporter gene (*PRH1* promoter driving *LUC*) was co-transformed with one or two of the constructs *35S*::*LBD16*, *35S*::*LBD18* and *35S*::*LBD29*. The empty vector pBI221 was used as negative control. Values shown as means±SE, three biological replicates in the experiment. *, **: means differ significantly (*P*<0.05, *P<*0.01) from the WT control.

The transcriptional response of *PRH1* to the loss-of-function and over-expression of certain LBDs implied that these transcription factors can also regulate *PRH1*. The *PRH1* promoter has the typical LBD protein binding elements including CGGCG and CACGTG (S6A Fig). Although the yeast one hybrid assay produced no direct evidence for the binding of LBD proteins to the *PRH1* promoter (S6B Fig), but the ChIP-qPCR assay did support the association of LBD16, LBD18 and LBD29 with the *PRH1* promoter (Fig 5A). Thus a transient expression assay in *A*. *thaliana* leaf protoplasts was used to investigate whether LBD16, LBD18 and/or LBD29 was able to influence the expression of the *PRH1pro*:*LUC* transgene. The outcome of co-expressing this construct with each of *35S*::*LBD16*, *35S*::*LBD18* or *35S*::*LBD29* was to enhance the expression of *PRH1pro*:*LUC* (Fig 5B). When a pair of effector transgenes (either *35S*::*LBD16* plus *35S*::*LBD18*, *35S*::*LBD18* plus *35S*::*LBD29* or *35S*::*LBD16* plus *35S*::*LBD29)* was introduced, the *PRH1pro*:*LUC* expression was even more strongly activated (Fig 5B). The overall conclusion was that the LBDs regulate *PRH1* expression by directly binding to its promoter.

### LBD regulated LR development is partially dependent on PRH1

**Fig 6:**
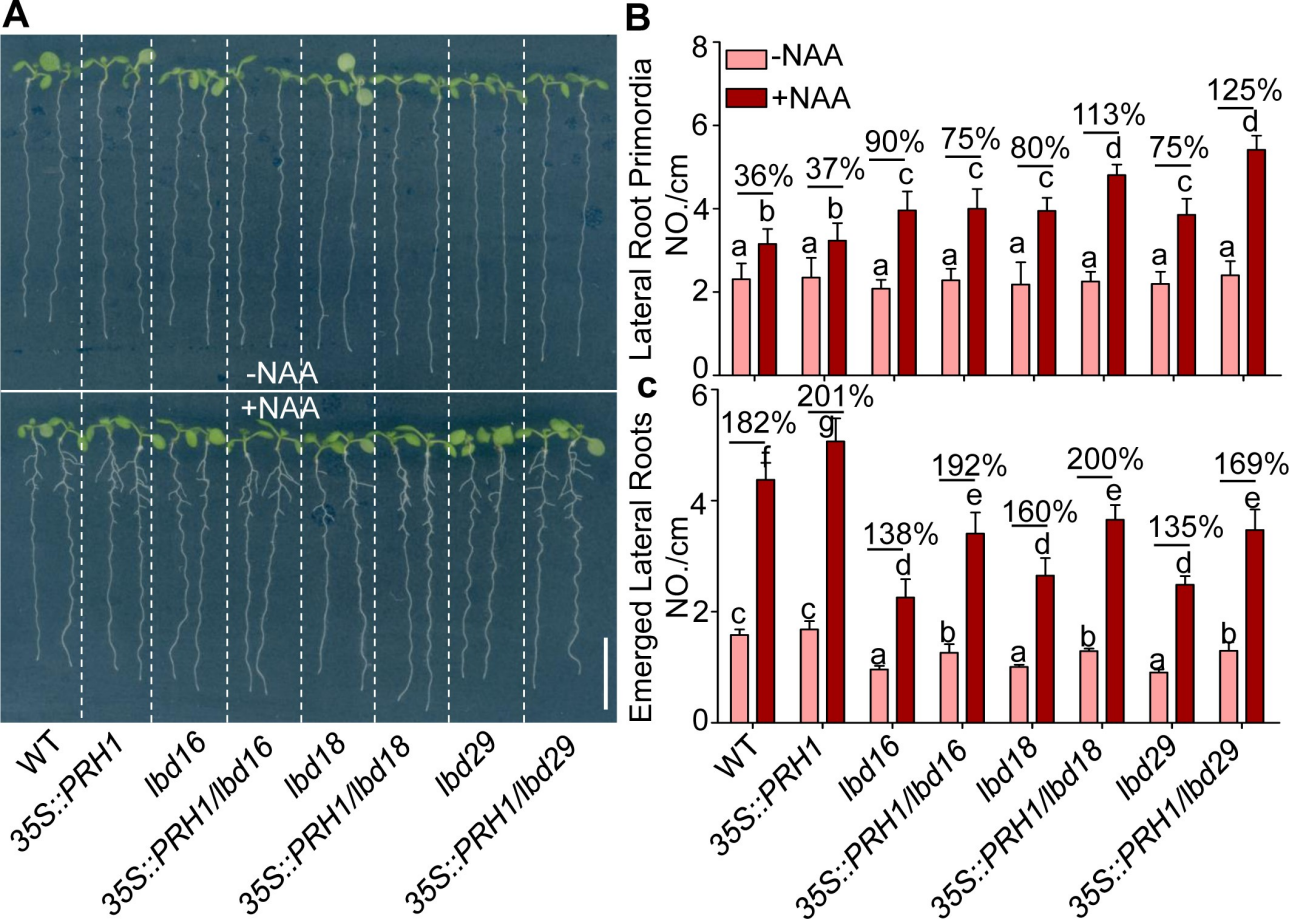
The over-expression of *PRH1* partially alleviates the defective development of lateral roots (LRs) in the *lbd* mutant. (A) Eight-day-old seedlings of WT, the mutants *lbd16*, *lbd18* and *lbd29*, and the transgenic lines harboring *35S*::*PRH1*. The upper panel shows the seedlings grown on 1/2 MS medium without NAA addition and the lower panel shows the seedlings grown on 1/2 MS medium containing 30 nM NAA. Scale bar: 1 cm. (B and C) Density of lateral root primordia (B) and emerged lateral roots (C) in eight-day-old seedlings grown on the 1/2 MS medium containing 30 nM NAA or not. The percentage values represent the increase with NAA treatment. The density was the ratio between lateral roots number and the length of primary root. NAA: naphthalene acetic acid. Data shown as means±SE, three biological replicates in the experiment, 20 plant seedlings for each repeat. Different letters atop the columns indicate significant (*P*<0.05) differences in abundance.

To further study if LBDs regulated *PRH1* transcription is involved into LBD-mediated LR development, we crossed *35S*::*PRH1* with *lbd16*, *lbd18* or *lbd29* mutants and analyzed LR phenotypes of the generated double mutants. Compared to *lbd16*, *lbd18*, *lbd29* single mutant plants, the generated *35S*::*PRH1/lbd16*, *35S*::*PRH1 lbd18* or *35S*::*PRH1/lbd29* double mutants have increased LR numbers, especially in the presence of auxin treatment (Fig 6), indicating that *PRH1* is involved into LBD-mediated LR development.

To determine the subcellular localization of PRH1, the cytosolic marker ADH-RFP [29] or plasma membrane maker ZmCRN-mCherry [30] were coexpressed with PRH1-GFP in N. benthamiana leaf cells (S7 Fig). The green signals from PRH1-GFP were merged with the red signals from ADH-RFP, indicating that PRH1 is localized in the cytosol. Given the LR phenotype of *prh1* mutants and its expression pattern, the expression levels of some cell wall formation related genes were detected in *prh1-1*. We found some *EXPANSIN* genes, including *EXP3 EXP8 EXP11* and *EXP14* which were all involved in cell wall loosening [12, 31], were down-regulated in *prh1* (S8 Fig). Therefore, PRH1 might regulate LR emergence by influencing the *EXPANSIN* gene expression.

**Fig 7:**
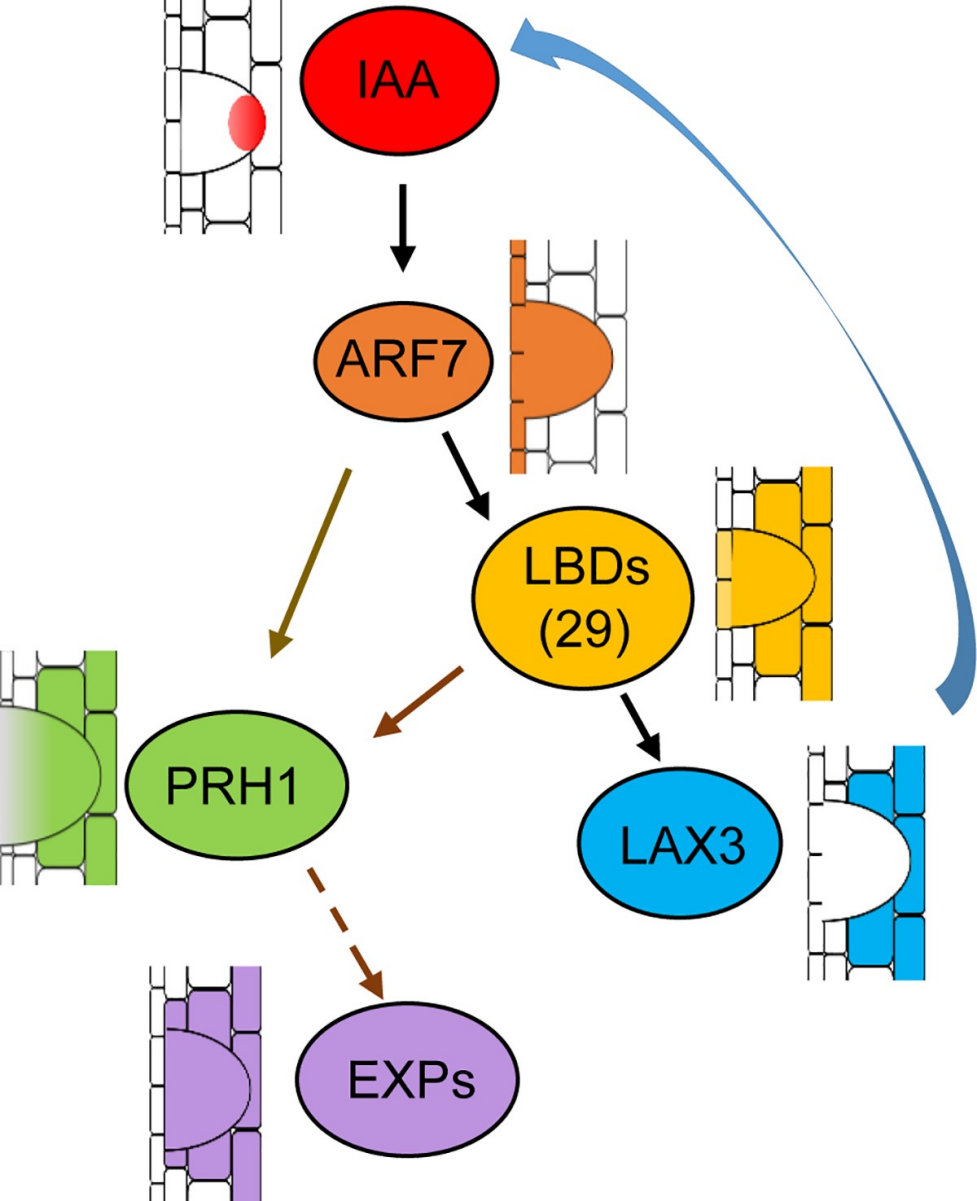
A working model of the regulation of LR development by PRH1. Auxin induced *PRH1* expression is dependent on both ARF7 and LBDs. LAX3, which acts downstream of LBD29, also indirectly regulates *PRH1* expression through controlling IAA accumulation in the epidermits and cortex. PRH1 may promote LR emergence by regulating the expression of *EXPANSIN* genes. *ARF7* is expressed in pericycle and primordium [33]; *LBD16* is expressed in the primordium at earlier stages [9]; *LBD18* and *LBD29* are expressed in the LR primordium and overlying tissues [27]; *LAX3* is expressed in the epidermis and cortex cells above the primordia [27]. The histological localization of PRH1 is in the cells overlying/surrounding the lateral root primordium. PRH1 might regulate LR emergence through influencing the expression of *EXPs* that affecting cell wall loosening.

According to this study together with the previous reports, a proposed model was given in Fig 7. PRH1 is involved in ARF7-LBD dependent auxin signaling pathway regulating lateral root development. PRH1 acts as a downstream gene of both ARF7 and LBDs by the direct transcriptional regulation and then affects the expression of some EXPANSIN genes. Meanwhile LAX3 acts downstream of LBD29 to control IAA accumulation in the epidermits and cortex [32], and thus influences auxin-induced *PRH1* expression indirectly.

## Discussion

### The product of the auxin-induced gene PRH1 controls LR formation

The role of auxin in LR development has been extensively investigated. During the initiation of LR, auxin signals are transduced through the two distinct AUX/IAA-ARFs modules IAA14-ARF7/ARF19 and IAA12-ARF5 [6, 33–36]. This process establishes LR founder cell identity, which is the initial step towards the formation of a LR. Auxin is also involved in LR emergence, since it controls the activation of *IDA*, *HAE* and *HSL2* which encode cell wall remodeling enzymes and of genes encoding various expansions in the endodermal cells which lie above the LR primordia [11, 15, 37]. Members of the *LBD* gene family encode transcription factors, characterized by their LOB domain [38], which act downstream of ARF7-mediated auxin signaling to control LR formation [13, 24–26].

Here, the *PR-1* homolog gene *PRH1* was identified as a novel target of ARF7-mediated auxin signaling. Otherwise we have compared our RNA-Seq data to the previous datasets of lateral root induction, recommend in Lewis et al [39] and Himanen et al [40]. Consistently, all these studies showed that the expression of *PRH1* was induced by auxin and the induction depended on ARF7. Although the over-expression of *PRH1* in a WT background was not associated with any LR phenotype, its over-expression in *arf7*, *lbd16*, *1bd18* or *lbd29* mutant backgrounds led to a partial rescue of the compromised LR phenotype, especially under auxin treatment (Figs 3 and 6). The more striking rescued LR formation by overexpression of *PRH1* in *lbd* mutants than that in *arf7* suggests additional factors other than LBDs act downstream of ARF7. The *PRH1* promoter directs its product primarily to the cells overlying/surrounding the lateral root primordium (as shown by the site of GUS expression in plants expressing a *PRH1pro*:*GUS* transgene), which is consistent with the proposed role of PRH1 in LR formation. Auxin treatment strongly induced the expression of the *PRH1pro*:*GUS* transgene in a WT (Fig 2A and 2B), but not in an *arf7* background (Fig 2C and 2D), which implied that the auxin-induced expression of *PRH1* must depend on the presence of a functional copy of *ARF7*. LBD proteins, especially LBD29, are also involved in the induction by auxin of *PRH1*. Experimentally, it was shown that both ARF7 and LBDs were able to bind to the *PRH1* promoter (Figs 4 and 5). As some of the LBD-binding and AuxRE elements close to each other in *PRH1* promoter (Fig 5A), it will be of future interest to investigate how ARF7 coordinates with LBDs to mediate auxin signaling and therefore to control the expression of *PRH1*. In addition, the auxin influx carrier LIKE-AUXIN3 (LAX3) which acts downstream of ARF7-LBD29 auxin signaling module to control LR emergence [27, 28], might be involved into auxin-induced *PRH1* expression through the regulation of local auxin transport and local auxin accumulations.

The homo- and heterodimerization of LBD proteins are thought to be an important determinant of LR formation [26, 41]. The AtbZIP59 transcription factor has been reported to form complexes with LBDs to direct auxin-induced callus formation and LR development [42]. All these results suggest that homodimerization and heterodimerization of signaling components are important in LR formation. Here, it was shown that *PRH1* was mainly expressed in the cells overlying the primordium (Fig 2) and there was a LR emergence defect in the *prh1* mutants. Several of the *EXPANSIN* genes were down-regulated in *prh1-1*(S8 Fig), indicating that PRH1 might regulate LR emergence through influencing cell wall loosening. Whether PRH1 regulates LR formation through a direct interaction with LBDs, AtZIP59 and other factors which have been shown to control LR development, is still an open question for the future.

## Materials and methods

### Plant materials and growing conditions

*A*. *thaliana* ecotype Col-0 was used as the wild type (WT), and all mutants and transgenes were constructed in a Col-0 background. The mutants comprised a set of T-DNA insert lines obtained from the *Arabidopsis* Biological Resource Center (ABRC, Columbus, OH, USA), namely *prh1-1* (GK_626H08), *prh1-2* (Salk_014249), *lbd16* (Salk_040739), *lbd18* (Salk_038125) [43] and *lbd29* (Salk_071133) [27], along with *arf7* (Salk_040394) [33]; *lax3* [28]; the transgenic lines were *Super*::*LBD29-GFP* [44], *35S*::*MYC-LBD16*, *35S*::*MYC-LBD18*, *35S*::*PRH1*, *35S*::*PRH1-GFP* (PRH1-GFP) and *PRH1pro*:*GUS*. Seeds were surface-sterilized by fumigation with chlorine gas for 4 hours, and then plated on solidified half strength Murashige and Skoog (MS) medium. All of the seeds were held for two days at 4°C and finally grown in a greenhouse delivering a 16 h photoperiod with a constant temperature of 22°C.

### Transgene constructs and the generation of transgenic lines

The 1,692 nt region upstream of the *PRH1* start cordon was used as the *PRH1* promoter, and was inserted, along with the *PRH1* coding sequence into a pENTR^TM^/SD/D-TOPO^TM^ plasmid (ThermoFisher). The construct was subsequently recombined with several Gateway destination vectors, namely PK7WG2 [45] for over-expression without any tags, PB7WGF2 for over-expression with an C-GFP tag, PGWB18 for over-expression with an N-Myc tag, PBI221 for over-expression, PKGWFS7.1 [45] for promoter analysis with C-GFP/GUS. Transgenesis was effected using the floral dip method [46], employing transgene constructs harbored by *Agrobacterium tumefaciens* strain GV3101.

### Microscopy

The PRH1 subcellular localization was detected in tobacco leaf cells. The *Agrobacterium tumefaciens* strain GV3101 containing *35S*::*PRH1-GFP* or the cytosolic marker ADH-RFP [29] or plasma membrane maker ZmCRN-mCherry [30] were incubated shaking until the OD_600_ reaching 0.6. Centrifuge the cells at 4000g for 5 minutes, then discard the supernatant and resuspend the pellet with induction buffer (0.01mol/L MES, 0.01Mol/L MgCl_2_, 150 μmol/L acetosyringone) to find OD = 0.4. Mix two kinds of agrobacterium up with a same volume and inject the mixture into 3 week old tobacco leaves. The transfected plants grown in the greenhouse for at least 48 h at 28°C before GFP and RFP caning. The fluorescent signals were visualized by using the LSM880 laser scanning confocal microscope (Zeiss). ZmCRN-mCherry and ADH-RFP signals were detected by using 561 nm laser excitation and 580–675 nm emission, PRH1-GFP signals were detected by using 561 nm laser excitation and 500–530 nm emission.

The 8-d-old seedlings of *PRH1pro*:*GUS* were used for GUS staining. The GUS staining assay followed Liu et al. [47]. GUS signal was photographed by using an Olympus BX53 microscope supported with an Olympus DP72 digital camera.

### Phenotype analysis

Eight-day-old seedlings were photographed using a Epson Perfection V800 scanner for the whole seedling images and the root length was measured by Image J software. To observe lateral root primordia clearly the seedlings were treated with a serials of solutions (1. 4% HCL, 20% methanol at 57°C for 15 minutes. 2. 7% NaOH, 60% ethanol at room temperature for 15 minutes. 3. 60% ethanol at room temperature for 10 minutes. 4. 40% ethanol at room temperature for 10 minutes. 5. 20% ethanol at room temperature for 10 minutes. 6. 10% ethanol at room temperature for 10 minutes. 7. 25% glycerol, 5% ethanol at room temperature for 10 minutes) before mounted in 50% glycerol on slides. Then the lateral root primordia and the emerged lateral roots were calculated using the Olympus BX53 microscope. The differential stages of lateral roots development have been described by Malamy and Benfey [1].

### RNA isolation and quantitative real time PCR (qPCR) analysis

RNA was extracted from the roots of eight-day-old seedlings (some of which had been treated with 10 μM naphthalene acetic acid (NAA), obtained from Sigma (St. Louis, MO, USA)) using a TIANGEN Biotech (Beijing) Co. RNA simple Total RNA kit (www.tiangen.com), following the manufacturer’s protocol. A 2 μg aliquot of the RNA was used as the template for synthesizing the first cDNA strand, using a Fast Quant RT Kit (TIANGEN Biotech (Beijing) Co.). Subsequent qPCRs were processed on an MyiQ Real-time PCR Detection System (Bio-Rad, Hercules, CA, USA), using the Super Real PreMix Plus SYBR Green reagent (TIANGEN Biotech (Beijing) Co.). Each sample was represented by three biological replicates, and each biological replicate by three technical replicates. The *AtACTIN2* (*At3g18780*) sequence was used as the reference. Details of the relevant primer sequences are given in S3 Table.

### RNA-Seq analysis

RNA submitted for RNA-seq analysis was isolated from the roots of eight-day-old WT and *arf7* seedlings (treated with the 10 μM naphthalene acetic acid (NAA) for 4 hours and the non-treated samples), using a RNeasy Mini Kit (Qiagen, Hilden, Germany). The RNA-seq analysis were processed following the methods given by Lv et al. [48]

### Chromatin immunoprecipitation coupled to quantitative PCR (ChIP-qPCR) analysis

DNA harvested from eight-day-old seedlings of WT and transgenic lines harboring either *35S*::*MYC-ARF7*, *35S*::*MYC-LBD16*, *35S*::*MYC-LBD18* (*ARF7*, *LBD16* and *LBD18* were cloned into PGWB18) or *Super*::*LBD29-GFP* [44, 49] then they were cross-linked in a solution containing 1% v/v formaldehyde. The ChIP procedure followed that given by Gendrel A-V et al. [50], and employed antibodies recognizing MYC and GFP (Abcam, UK). The immune-precipitated DNA and total input DNA were analyzed by qPCR. The enrichment in WT and transgenic plants was measured as a ratio of the bound promoter fragments over total input respectively. Details of the relevant primers are given in S3 Table.

### Yeast one-hybrid (Y1H) assays

Y1H assays were carried out using the Matchmaker Gold yeast one hybrid Library Screening System (Biosciences Clontech, Palo Alto, CA, USA). The *PRH1* promoter was inserted into the *pAbAi* vector, and the bait vector (*PRH1*_*pro*_*-pAbAi*) was linearized, restricted by *Bbs*I, and introduced into the Y1H Goldeast strain along with the prey vector AD-ARF7. Transformants were grown on SD/-Leu-Ura dropout plates containing different concentrations of aureobasidin A (CAT#630499, Clontech, USA). The primers used for generating the various constructs are listed in S3 Table.

### Dual-luciferase transient expression assay in *A*. *thaliana* protoplasts

Protoplasts were isolated from three week old WT leaves following Yoo et al. [51]. The ligation of the *PRH1* promoter to pGreen0800-LUC was realized by enzyme digestion and ligation [52]. The primers used for this procedure are listed in S3 Table. The *PRH1pro*:*LUC* reporter plasmid was transferred into Arabidopsis protoplast cells with one or more effector plasmids (*ARF7*, *LBD16*, *LBD18*, *LBD29* –PBI221); the empty vector PBI221 was used as the negative control. The dual-luciferase assay kit (Promega, USA) allows for the quantification of both firefly and renilla luciferase activity. Signals were detected with a Synergy 2 multimode micro-plate (Centro LB960, Berthold, Germany).

### Generation of *prh1-3* using CRISPR technology

The experimental method was described by Yan et al [20]. The *Arabidopsis* plants Columbia-0 ecotype were used as wild type (WT) and were transformed with the CRISPR construct by floral dipping. The *prh1-3* plants were identified at the T2 stage. The target sequence is presented in S3 Table.

### Statistical analysis

Tests were applied to the data to check for normality and homogeneity before attempting an analysis of the variation. The data were analyzed using routines implemented in Prism 5 software (GraphPad Software). The Students’ *t-*test was applied to compare pairs of means. Values have been presented in the form means±SE; the significance thresholds were 0.05 (*), 0.01 (**) and 0.001 (***). Comparisons between multiple means were made using a one-way analysis of variance and Tukey’s test.

### Accession numbers

Sequence data referred to in this paper have been deposited in both the *Arabidopsis* Genome Initiative and the GenBank/EMBL databases under the accession numbers: *ARF7* (At5g20730, NP_851046), *PRH1* (At2g19990, NP_179589), *LBD16* (*At2g42430*, NP_565973), *LBD18* (At2g45420, NP_850436), *LBD29* (At3g58190, NP_191378), *LAX3* (At1g77690, NP_177892). The RNA-seq data are available in the Gene Expression Omnibus database under accession number GSE122355.

## Supporting information

S1 Fig Transcriptomic analysis of WT and *arf7* roots before and after auxin treatment. (A) Numbers of DEGs in *arf7* roots compared to WT with auxin treatment (treated with 10 μM naphthalene acetic acid (NAA) for 4 hours) or not. (B) Selected candidate genes from both the up-regulated DEGs in WT by auxin and down-regulated genes in *arf7* (the bigger purple circle), and the 8 targets involved in lateral root development further refined by *Arabidops is* eFP Browser from the 23 selected candidates (the smaller yellow circle). (C) The expression pattern and change folds of 8 targets at the site of lateral roots initiation in WT and *slr-1* under the condition of auxin treatment or not.
(TIF)

S2 Fig *PRH1* responses to auxin treatment. (A) Phylogenetic tree of PRH1 in *Arabidopsis*. Numbers in green represent bootstrap values. The key genes *PR1* and *PRH1* were marked in red. (B) The induction of *PRH1* by auxin (10 μM NAA) was monitored in wild-type (Col-0) by qPCR. Values represent averages of three biological replicates in the experiment, and the total RNA was extracted from the primary roots of about 100 seedlings for each repeat. NAA: naphthalene acetic acid. Error bars represent SE. (C) Expression pattern of *PRH1* in intact seedlings before (left) and after (right) a 4 hours exposure to 10 μM NAA. GUS signals appear blue. GUS: β-glucuronidase. Bar: 0.5 cm.
(TIF)

S3 Fig Lateral roots (LRs) development phenotype analysis and the transcription level of *PRH1* in wild type (WT), *prh1* mutants and its over-expression lines. (A) A map of the T-DNA insertion and the frameshift mutant of *prh1* on chromosome (upper panel), and the single base insertion site in the coding sequence (lower panel). The orange line represents the promoter region, the black box represents the coding region and the blue lines represent the insertion sites. (B) Latral root (LR) number per centimeter (cm) along the primary root of the PRH1 over-expression lines. Values shown as mean±SE, three biological replicates in the experiment, 20 plant seedlings for each repeat. (C) The *PRH1* relative transcript aboundance in the primary roots of WT, *prh1* mutants and plants over-expressing *PRH1*. Three biological replicates in the experiment. Total mRNA was extracted from the primary roots of about 100 seedlings for each repeat. Data represent means±SE. **, ***: means differ significantly (P<0.01, P<0.001) from the WT control.
(TIF)

S4 Fig PRH1 could complement the defects of Lateral roots (LRs) formation in *prh1* mutants. (A) The 8-day-old seedlings of WT, *prh1-1* and RH1pro-PRH1 transgenetic lines in both WT and the *prh1-1* background. Bar: 1 cm. (B) LR phenotyping was achieved by synchronizing lateral root formation with a gravistimulus for 18 h and 42 h. Primordia stages from I to VII were based on the classification by Malamy and Benfey [1] and the data were analysed from 20 seedlings. (C) Density of primordia at given stages. *,**: means differ significantly (P<0.05, P<0.01) from the WT control. (D) The LR density of WT, *prh1-1* and the transgenetic lines. LRP: lateral root primordia. LRE: emerged lateral root, LRT: total lateral roots including the LRP and LRE. Data shown as means±SE, three biological replicates in the experiment, twenty plant seedlings for each repeat. The asterisks indicate means which differ significantly (*P*<0.05) from one another.
(TIF)

S5 Fig The transcription of *PRH1* is regulated by LBD16, LBD18, LBD29 and LAX3. (A) Transcription level of *PRH1* in the roots of WT, *lbd16*, *lbd18* and *lbd29* seedlings exposed to 10 μM naphthalene acetic acid (NAA) for 4 hours (orange) or not treated (brown) before extracting total mRNA. Different letters atop the columns indicate significant (*P*<0.05) differences in abundance. (B) Transcription level of *PRH1* is enhanced in each of the *LBD16*, *18* and *29* over-expression lines. Data represent means±SE, three biological replicates in the experiment **, ***: means differ significantly (P<0.01, P<0.001) from the WT. (C) *LAX3* expression in *prh1-1* in response to auxin. Eight-day-old seedlings were incubated with 10 μM naphthalene acetic acid (NAA) for 4 hours. Total mRNA were extracted from the primary roots and subjected to qPCR. Data are the means±SE, three independent biological replications. Different letters atop the columns indicate significant (*P*<0.05) differences in abundance. (D) *PRH1* expression in *lax3* in response to auxin. Eight-day-old seedlings were incubated with 10 μM naphthalene acetic acid (NAA) for 4 hours. Total mRNA were extracted from the primary roots and subjected to qPCR. Data are the means±SE, three independent biological replications. Different letters atop the columns indicate significant (*P*<0.05) differences in abundance.
(TIF)

S6 Fig The interaction between LBDs and *PRH1* promoter in yeast one-hybrid. (A) Structure of the *PRH1* promoter, showing the putative binding motifs of LBD18 (red square) [12] and LBD29 (yellow square) [44]. (B) Yeast one-hybrid binding assay containing the interaction between LBD16, LBD18, LBD29 and *PRH1* promoter.
(TIF)

S7 Fig The subcellular localization of PRH1. (A and B) Laser-scanning confocal image of PRH1-GFP fusion protein transiently expressed in *N*. *benthamian*a leaf cells with ADH-RFP as a cytosolic maker [29] (A) or ZmCRN-mCherry as a plasma membrane maker [30] (B). GFP: green fluorescent protein. RFP: red fluorescent protein. Scale bar: 20 μm.
(TIF)

S8 Fig Expression analysis of cell wall formation related genes in *prh1-1*. Eight-day-old seedlings of WT and the *prh1-1* mutant were used in this study. Total mRNA were extracted from the primary roots and subjected to qPCR. Data are the means±SE, three independent biological replications. The asterisk means differ significantly (P<0.05) from the WT.
(TIF)

S1 Table The raw data of gene expression based on the comparison between the four datasets. (XLSX)

S2 Table Gene differential expression filter data according to the comparison between the four datasets. (XLSX)

S3 Table Primers used in this study. (XLSX)
