# Correction: PRH1 mediates ARF7-LBD dependent auxin signaling to regulate lateral root development in *Arabidopsis thaliana*

**DOI:** 10.1371/journal.pgen.1010125

**Published:** 2022-03-15

**Authors:** Feng Zhang, Wenqing Tao, Ruiqi Sun, Junxia Wang, Cuiling Li, Xiangpei Kong, Huiyu Tian, Zhaojun Ding

There is an error in panel D of Fig 4. The authors have provided a corrected version of Fig 4 here.

**Fig 4:**
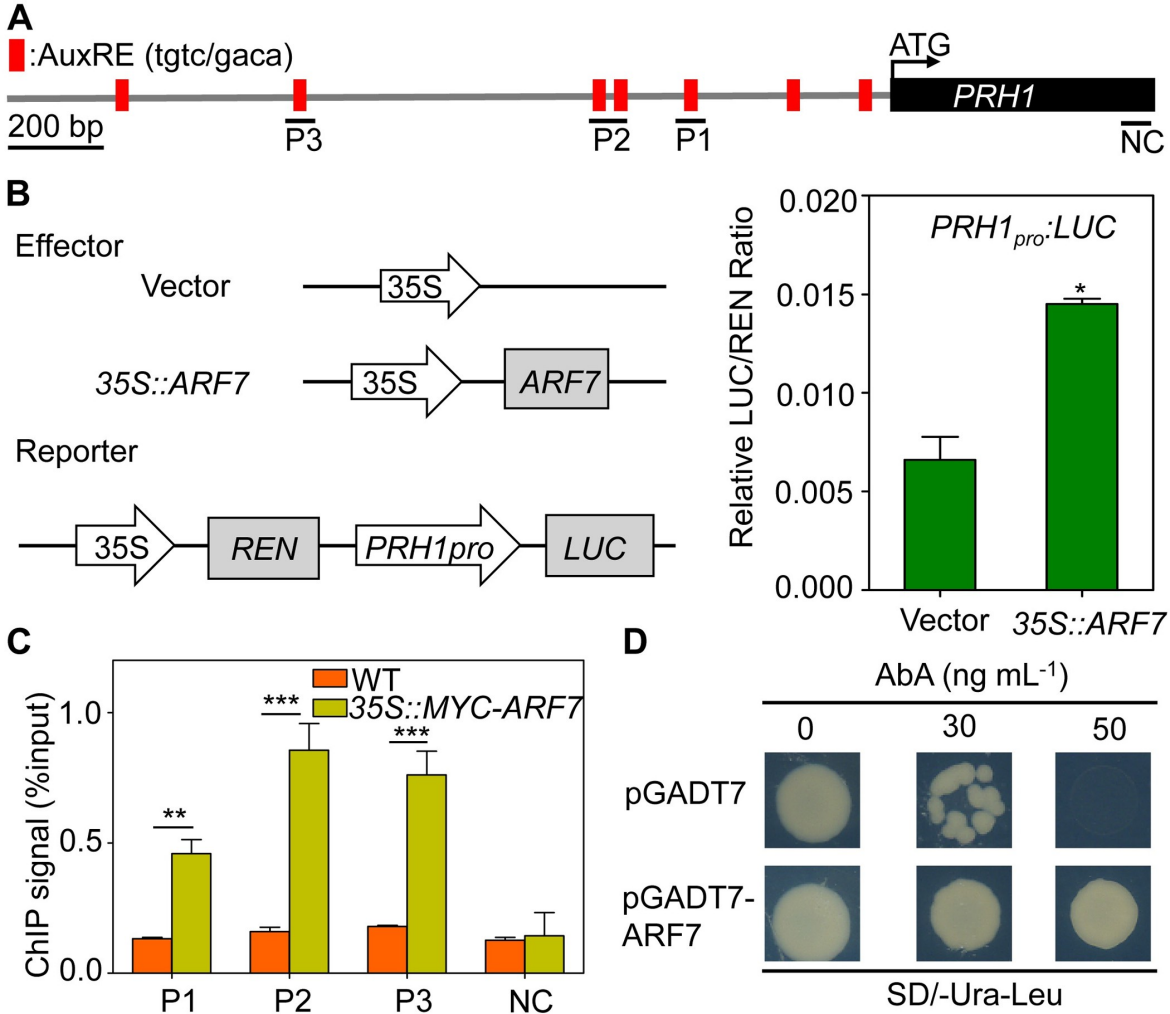
*PRH1* is regulated by *ARF7* at the transcriptional level. (A) Structure of *PRH1* promoter and the fragments used in the CHIP-qPCR assay. AuxREs are indicated by red squares, and black lines show the promoter regions containing the AuxREs used in this assay. NC: negative control. AuxREs: auxin response elements. (B) ARF7 transactivates the *PRH1* promoter in *A*. *thaliana* leaf protoplasts. The left hand panel is a schematic of the effector (*35S*::*ARF7*) and reporter (*PRH1pro*:*LUC*) constructs. The empty vector pBI221 was used as a negative control; the right hand panel shows the ratio of ARF7 drived *LUC* and the empty vector (negative control) to 35S promoter drived *REN* respectively. LUC: firefly luciferase activity, REN: renilla luciferase activity. Values shown as means±SE, three biological replicates in the experiment. *: means differ significantly (*P*<0.05) from the negative control. (C) ARF7 is associated with the *PRH1* promoter according to a CHIP-qPCR assay. Chromatin isolated from a plant harboring *35S*::*MYC-ARF7* and a WT mock control was immunoprecipitated with anti-MYC antibody following the amplification of regions P1, P2 and P3. The coding region segment NC was used as the negative control. The ChIP signal represents the ratio of bound promoter fragments (P1-P3) after immunoprecipitation to total input without immunoprecipitation. Values shown as means±SE, three biological replicates in the experiment. **, ***: means differ significantly (*P*<0.01, *P<*0.001) from the WT control. (D) Physical interaction of ARF7 with the *PRH1* promoter according to a Y1H assay. The plasmid pGADT7-ARF7 was introduced into Y1H Gold cells harboring the reporter gene *PRH1pro*:*AbAr* and the cells were grown on SD/-Ura-Leu medium in the presence of 30 or 50 ng/mL aureobasidin A (AbA). The empty vector pGADT7 was used as a negative control.
