# Supplementary figures and images for: PRH1 mediates ARF7-LBD dependent auxin signaling to regulate lateral root development in Arabidopsis thaliana
Source: PLoS Genet. 2020 Feb 7;16(2):e1008044. doi: 10.1371/journal.pgen.1008044 (PMC7006904; doi:10.1371/journal.pgen.1008044)

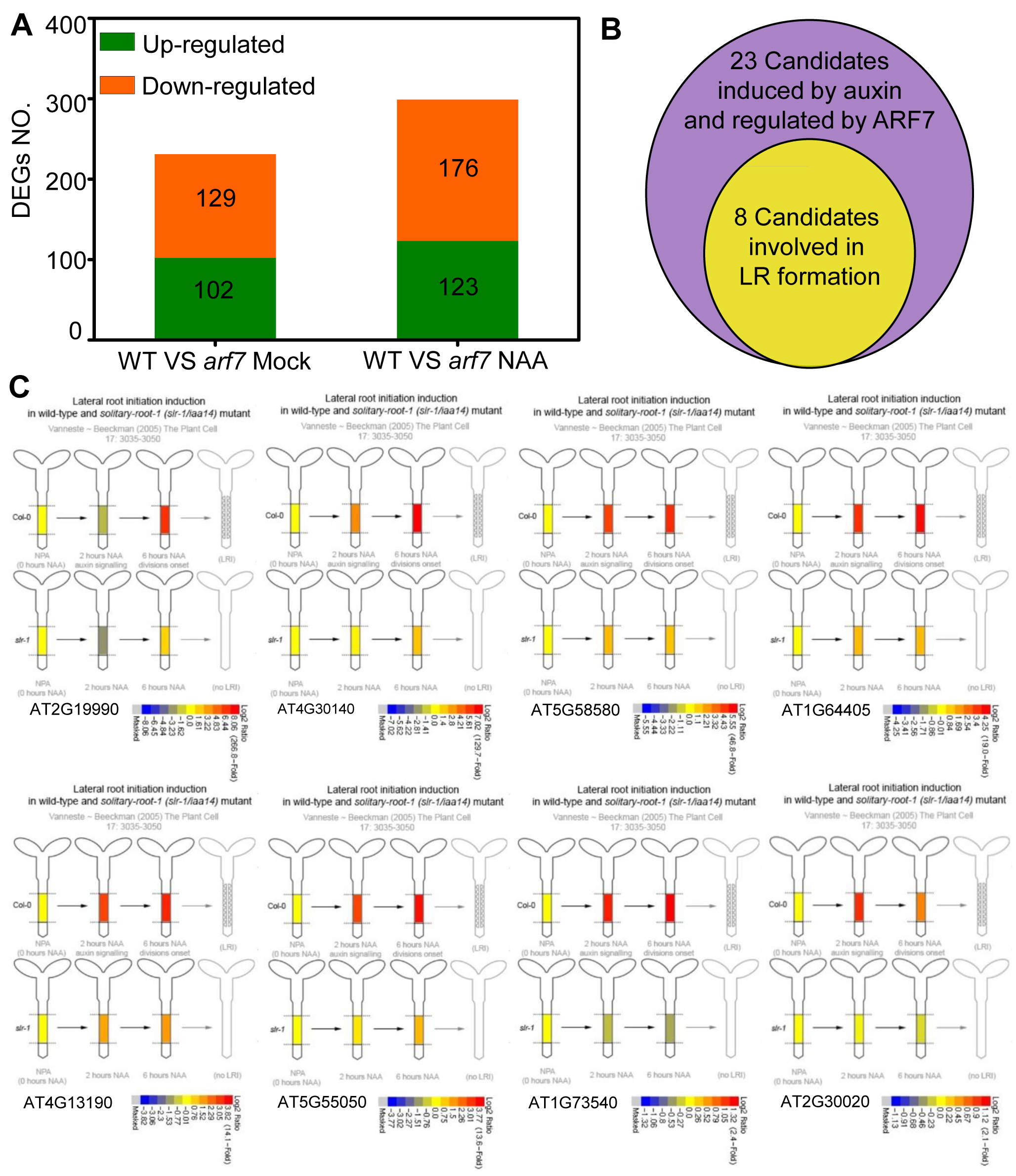

Supplement: S1 Fig — (A) Numbers of DEGs in arf7 roots compared to WT with auxin treatment (treated with 10 μM naphthalene acetic acid (NAA) for 4 hours) or not. (B) Selected candidate genes from both the up-regulated DEGs in WT by auxin and down-regulated genes in arf7 (the bigger purple circle), and the 8 targets involved in lateral root development further refined by Arabidops is eFP Browser from the 23 selected candidates (the smaller yellow circle). (C) The expression pattern and change folds of 8 targets at the site of lateral roots initiation in WT and slr-1 under the condition of auxin treatment or not. (TIF) [file pgen.1008044.s001.tif]

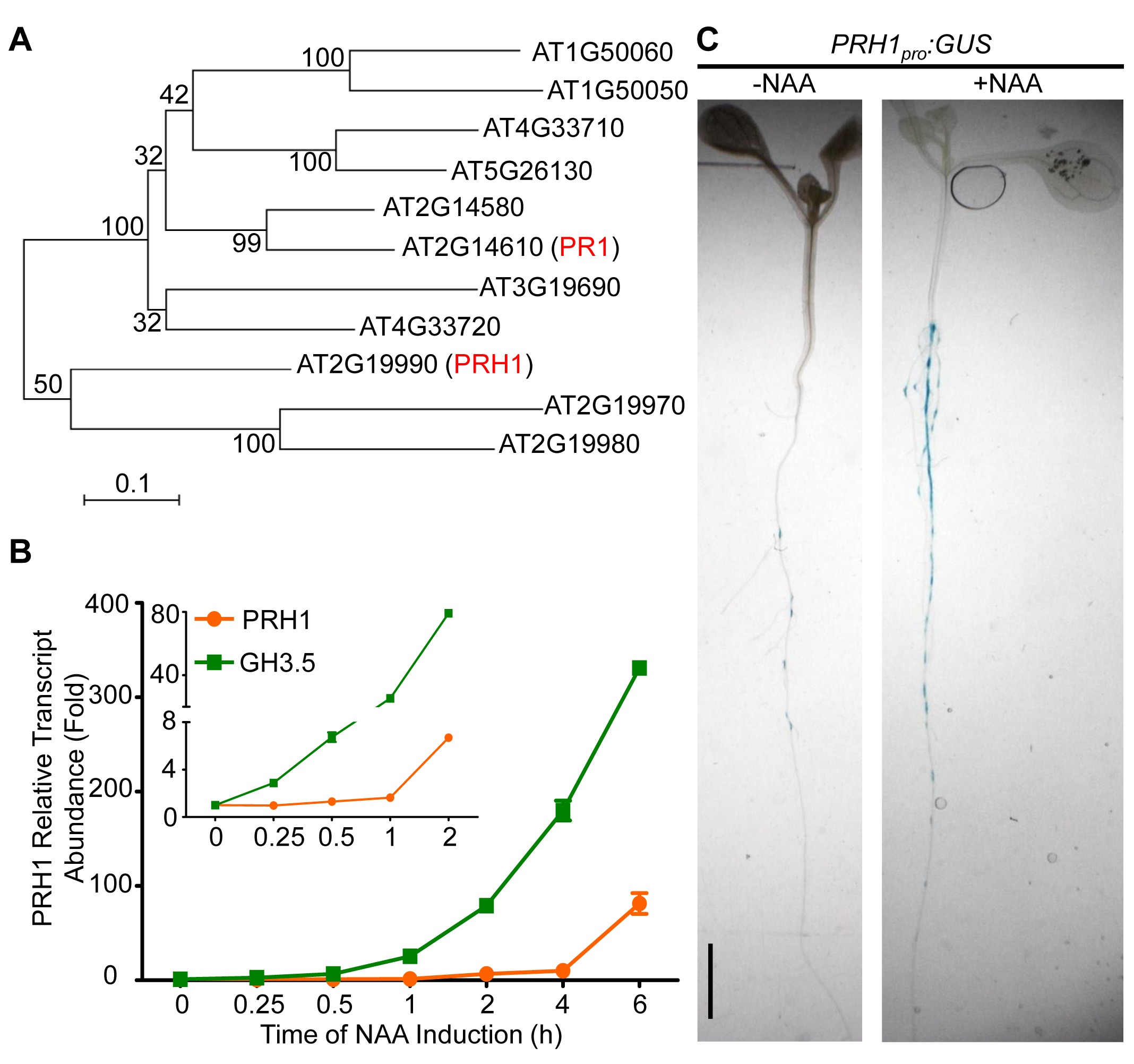

Supplement: S2 Fig — (A) Phylogenetic tree of PRH1 in Arabidopsis. Numbers in green represent bootstrap values. The key genes PR1 and PRH1 were marked in red. (B) The induction of PRH1 by auxin (10 μM NAA) was monitored in wild-type (Col-0) by qPCR. Values represent averages of three biological replicates in the experiment, and the total RNA was extracted from the primary roots of about 100 seedlings for each repeat. NAA: naphthalene acetic acid. Error bars represent SE. (C) Expression pattern of PRH1 in intact seedlings before (left) and after (right) a 4 hours exposure to 10 μM NAA. GUS signals appear blue. GUS: β-glucuronidase. Bar: 0.5 cm. (TIF) [file pgen.1008044.s002.tif]

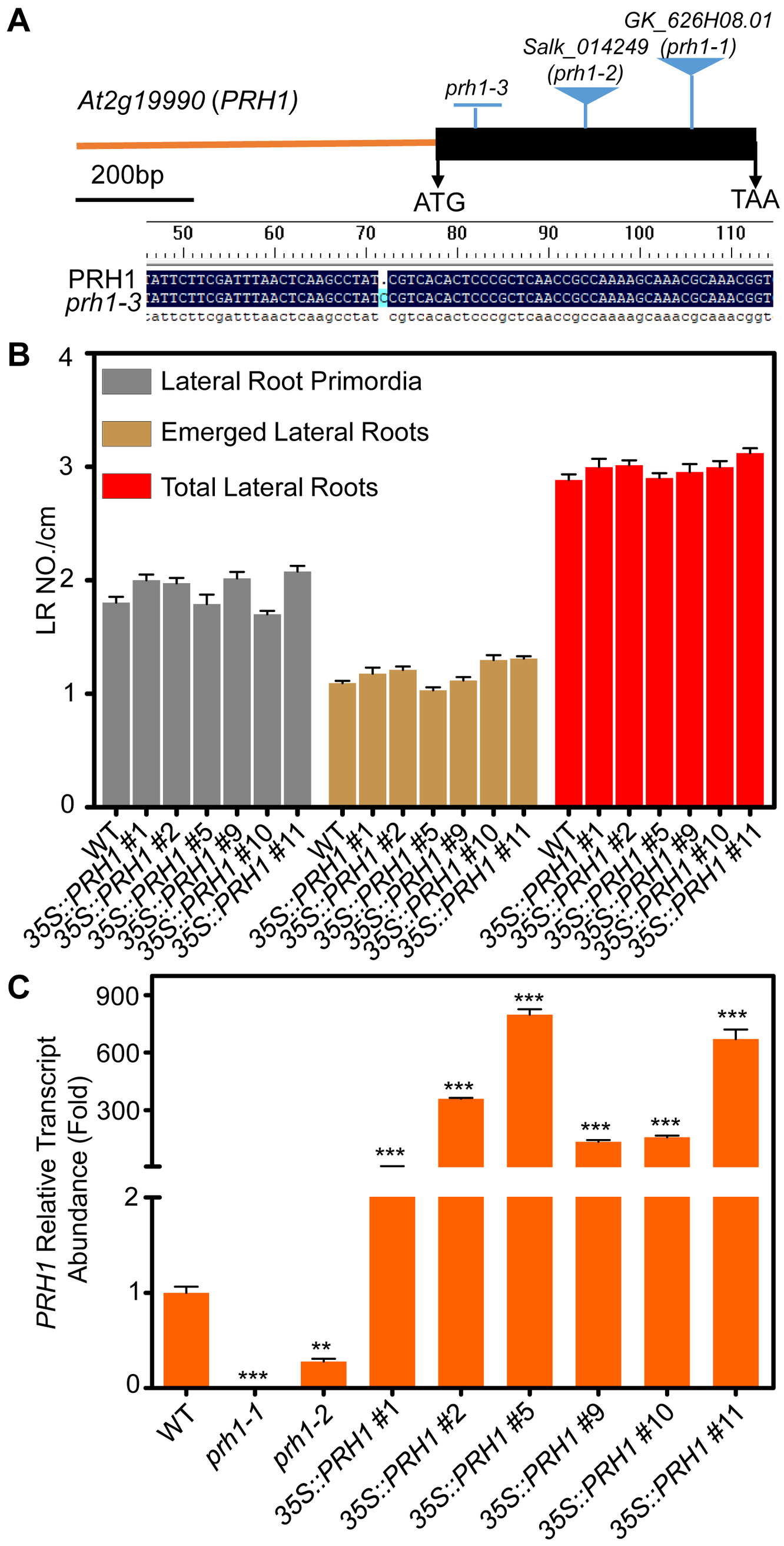

Supplement: S3 Fig — (A) A map of the T-DNA insertion and the frameshift mutant of prh1 on chromosome (upper panel), and the single base insertion site in the coding sequence (lower panel). The orange line represents the promoter region, the black box represents the coding region and the blue lines represent the insertion sites. (B) Latral root (LR) number per centimeter (cm) along the primary root of the PRH1 over-expression lines. Values shown as mean±SE, three biological replicates in the experiment, 20 plant seedlings for each repeat. (C) The PRH1 relative transcript aboundance in the primary roots of WT, prh1 mutants and plants over-expressing PRH1. Three biological replicates in the experiment. Total mRNA was extracted from the primary roots of about 100 seedlings for each repeat. Data represent means±SE. **, ***: means differ significantly (P<0.01, P<0.001) from the WT control. (TIF) [file pgen.1008044.s003.tif]

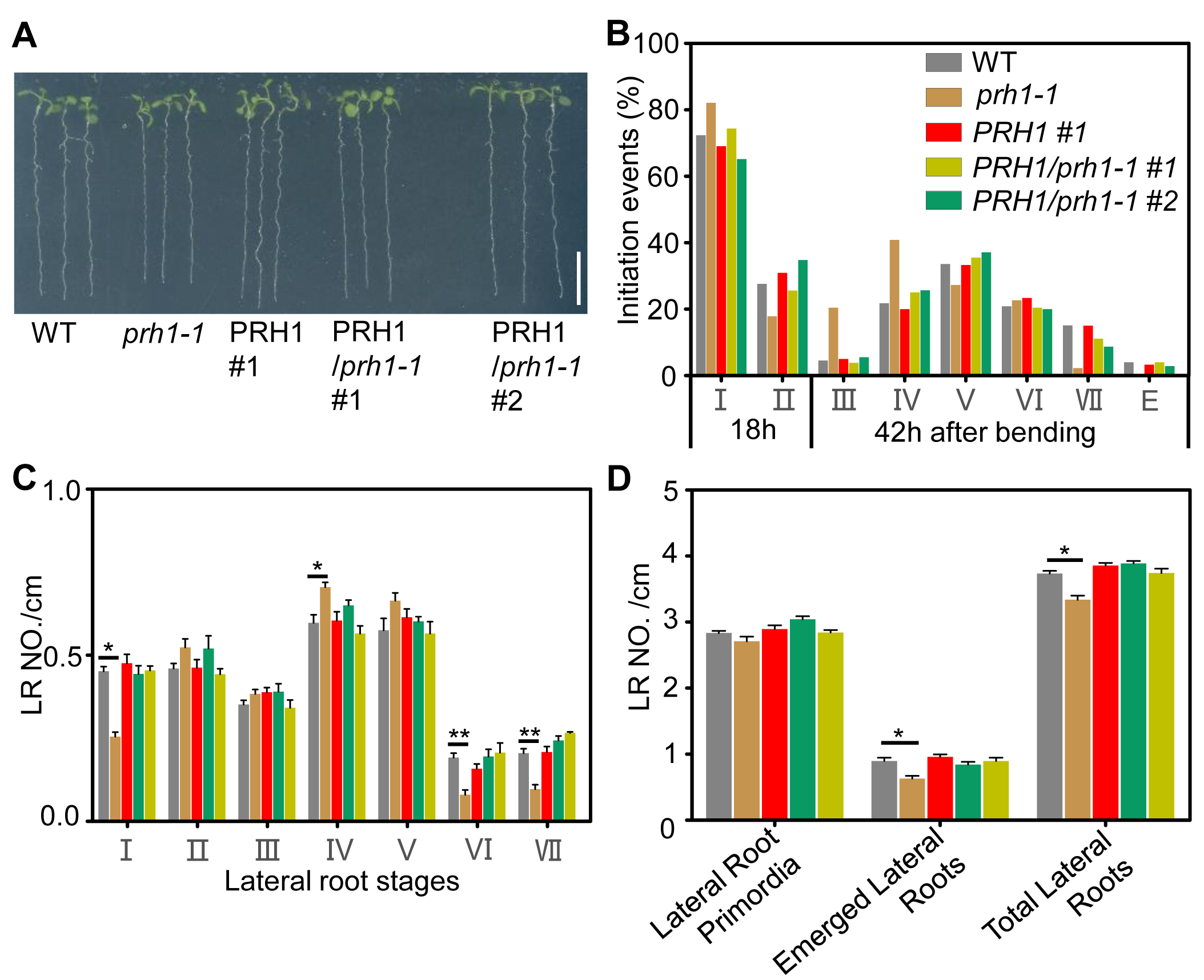

Supplement: S4 Fig — (A) The 8-day-old seedlings of WT, prh1-1 and RH1pro-PRH1 transgenetic lines in both WT and the prh1-1 background. Bar: 1 cm. (B) LR phenotyping was achieved by synchronizing lateral root formation with a gravistimulus for 18 h and 42 h. Primordia stages from I to VII were based on the classification by Malamy and Benfey [1] and the data were analysed from 20 seedlings. (C) Density of primordia at given stages. *,**: means differ significantly (P<0.05, P<0.01) from the WT control. (D) The LR density of WT, prh1-1 and the transgenetic lines. LRP: lateral root primordia. LRE: emerged lateral root, LRT: total lateral roots including the LRP and LRE. Data shown as means±SE, three biological replicates in the experiment, twenty plant seedlings for each repeat. The asterisks indicate means which differ significantly (P<0.05) from one another. (TIF) [file pgen.1008044.s004.tif]

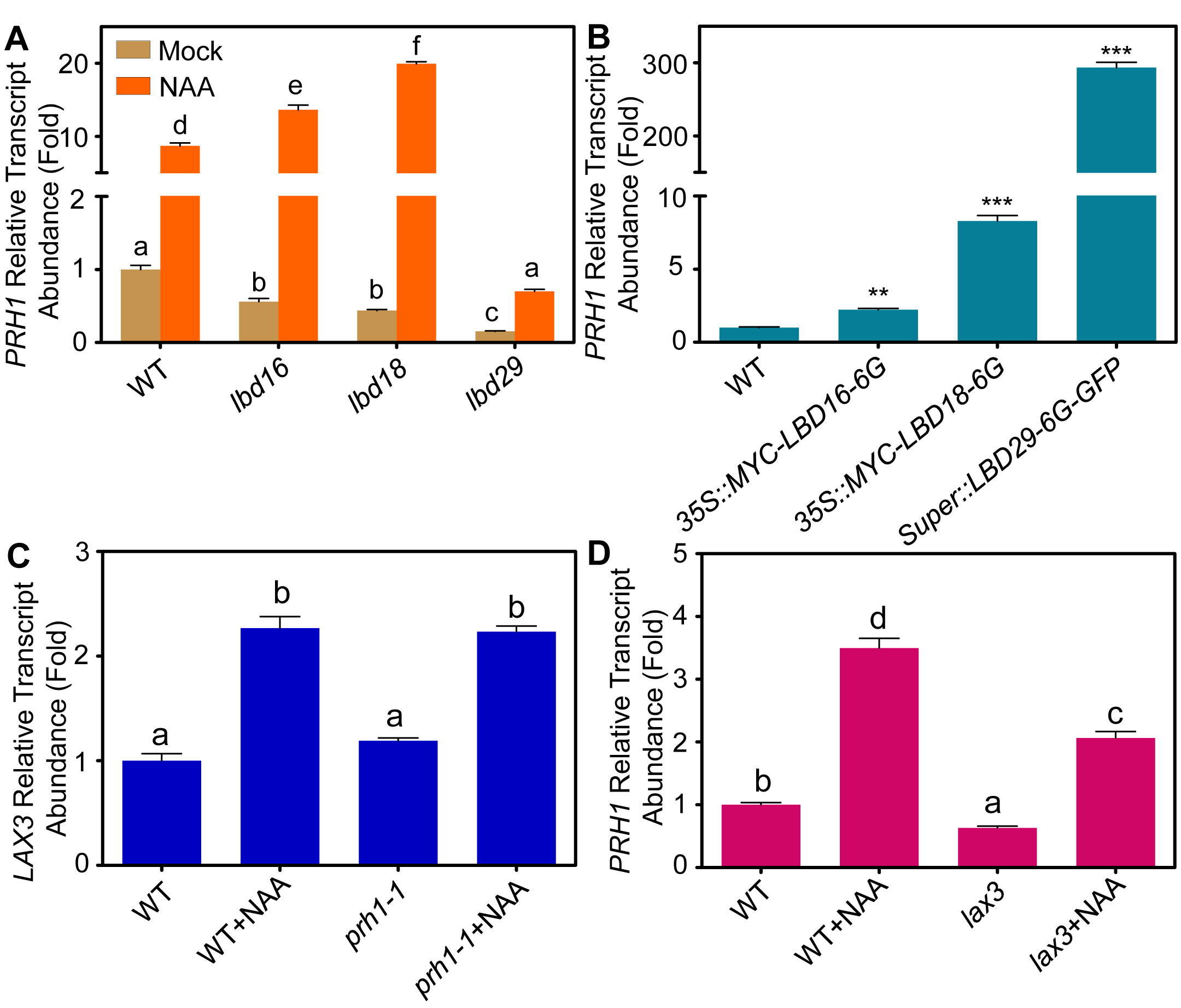

Supplement: S5 Fig — (A) Transcription level of PRH1 in the roots of WT, lbd16, lbd18 and lbd29 seedlings exposed to 10 μM naphthalene acetic acid (NAA) for 4 hours (orange) or not treated (brown) before extracting total mRNA. Different letters atop the columns indicate significant (P<0.05) differences in abundance. (B) Transcription level of PRH1 is enhanced in each of the LBD16, 18 and 29 over-expression lines. Data represent means±SE, three biological replicates in the experiment **, ***: means differ significantly (P<0.01, P<0.001) from the WT. (C) LAX3 expression in prh1-1 in response to auxin. Eight-day-old seedlings were incubated with 10 μM naphthalene acetic acid (NAA) for 4 hours. Total mRNA were extracted from the primary roots and subjected to qPCR. Data are the means±SE, three independent biological replications. Different letters atop the columns indicate significant (P<0.05) differences in abundance. (D) PRH1 expression in lax3 in response to auxin. Eight-day-old seedlings were incubated with 10 μM naphthalene acetic acid (NAA) for 4 hours. Total mRNA were extracted from the primary roots and subjected to qPCR. Data are the means±SE, three independent biological replications. Different letters atop the columns indicate significant (P<0.05) differences in abundance. (TIF) [file pgen.1008044.s005.tif]

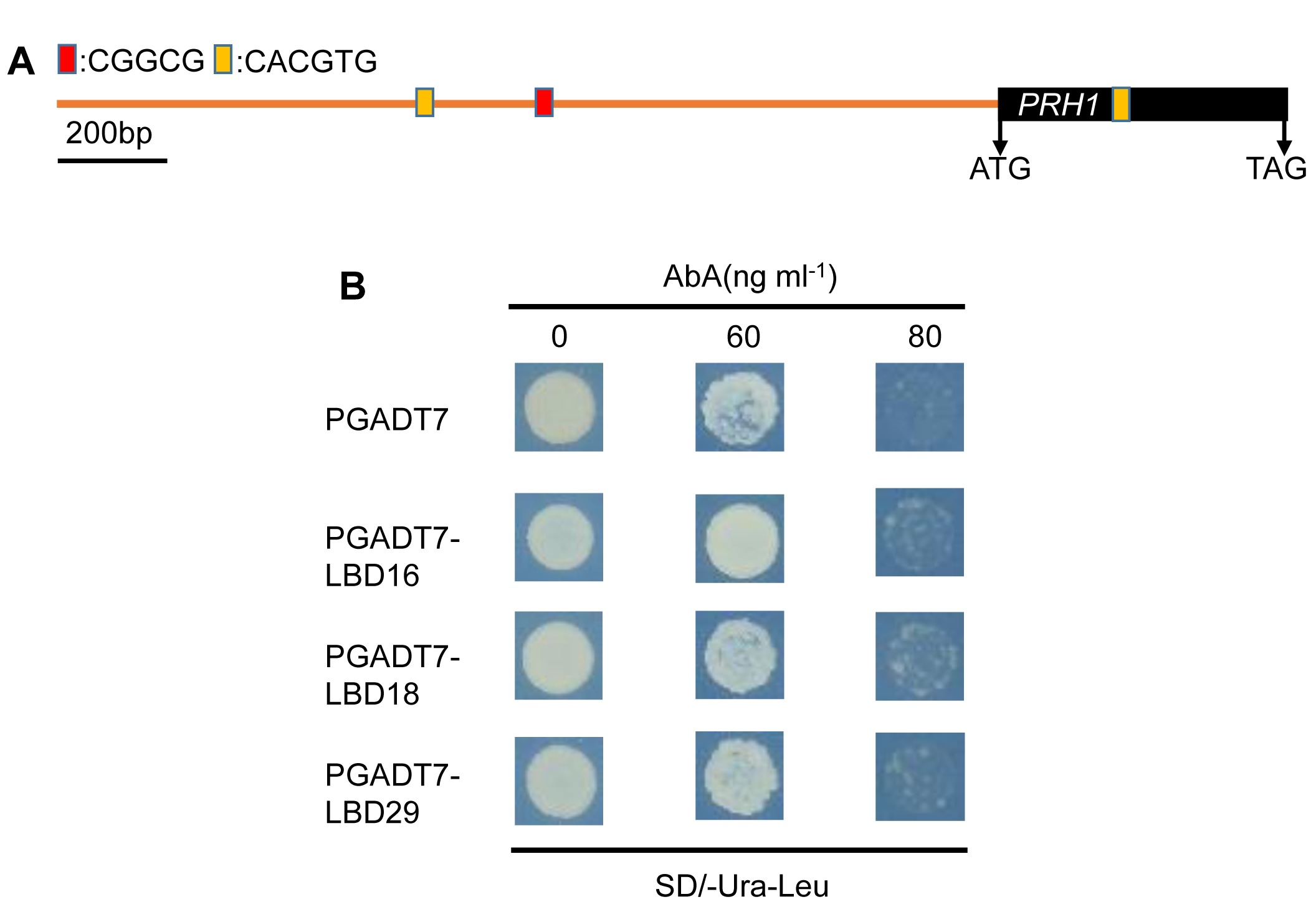

Supplement: S6 Fig — (A) Structure of the PRH1 promoter, showing the putative binding motifs of LBD18 (red square) [12] and LBD29 (yellow square) [44]. (B) Yeast one-hybrid binding assay containing the interaction between LBD16, LBD18, LBD29 and PRH1 promoter. (TIF) [file pgen.1008044.s006.tif]

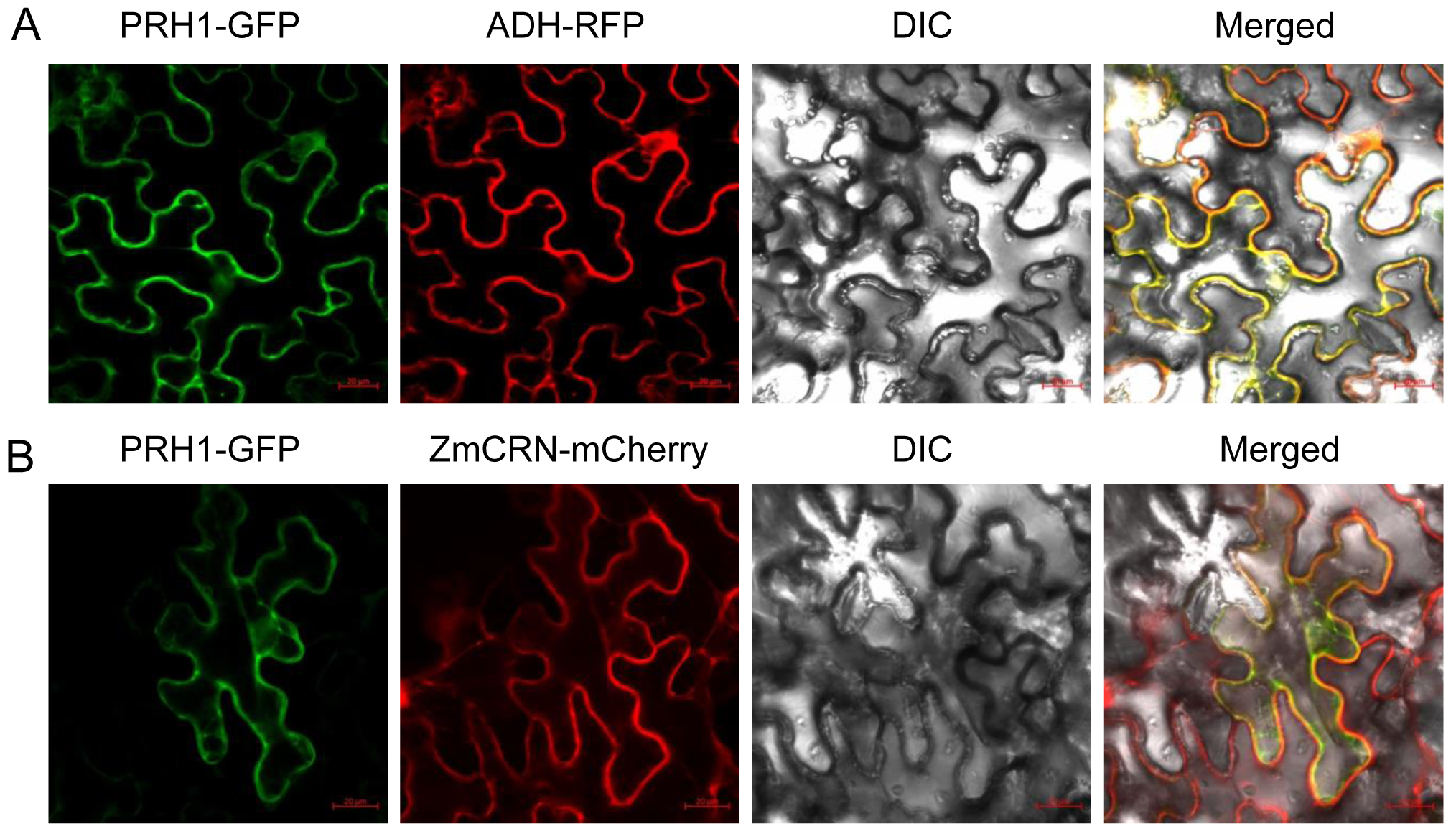

Supplement: S7 Fig — (A and B) Laser-scanning confocal image of PRH1-GFP fusion protein transiently expressed in N. benthamiana leaf cells with ADH-RFP as a cytosolic maker [29] (A) or ZmCRN-mCherry as a plasma membrane maker [30] (B). GFP: green fluorescent protein. RFP: red fluorescent protein. Scale bar: 20 μm. (TIF) [file pgen.1008044.s007.tif]

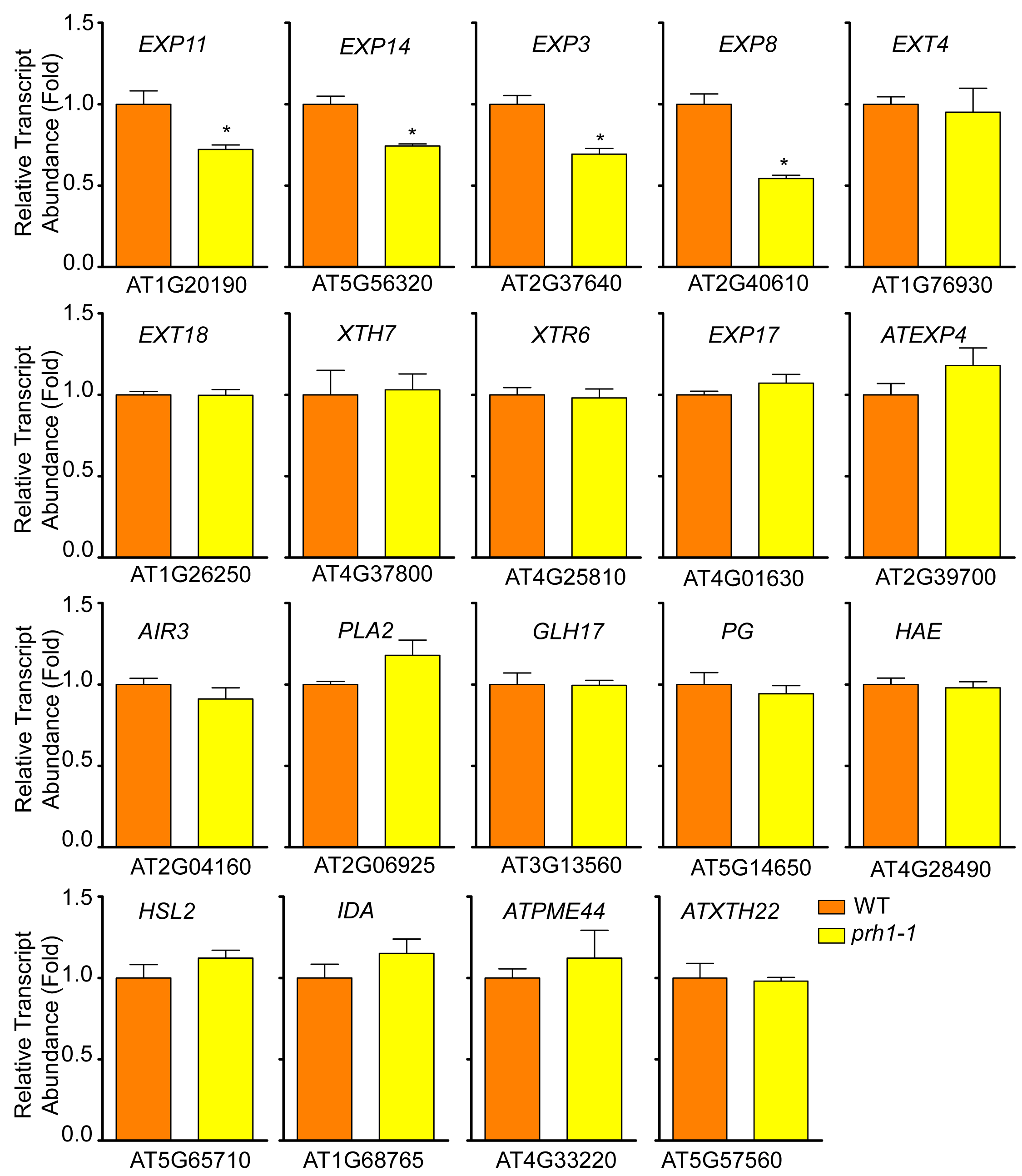

Supplement: S8 Fig — Eight-day-old seedlings of WT and the prh1-1 mutant were used in this study. Total mRNA were extracted from the primary roots and subjected to qPCR. Data are the means±SE, three independent biological replications. The asterisk means differ significantly (P<0.05) from the WT. (TIF) [file pgen.1008044.s008.tif]
